# Quantum interference enables constant-time quantum information processing

**DOI:** 10.1126/sciadv.aau9674

**Published:** 2019-07-19

**Authors:** M. Stobińska, A. Buraczewski, M. Moore, W. R. Clements, J. J. Renema, S. W. Nam, T. Gerrits, A. Lita, W. S. Kolthammer, A. Eckstein, I. A. Walmsley

**Affiliations:** 1 Institute of Theoretical Physics, Faculty of Physics, University of Warsaw, ul. Pasteura 5, 02-093 Warsaw, Poland.; 2 Clarendon Laboratory, University of Oxford, Parks Road, Oxford OX1 3PU, UK.; 3 Complex Photonic Systems (COPS), MESA+ Institute for Nanotechnology, University of Twente, P.O. Box 217, 7500 AE Enschede, Netherlands.; 4 National Institute of Standards and Technology, 325 Broadway, Boulder, CO 80305, USA.

## Abstract

It is an open question how fast information processing can be performed and whether quantum effects can speed up the best existing solutions. Signal extraction, analysis, and compression in diagnostics, astronomy, chemistry, and broadcasting build on the discrete Fourier transform. It is implemented with the fast Fourier transform (FFT) algorithm that assumes a periodic input of specific lengths, which rarely holds true. A lesser-known transform, the Kravchuk-Fourier (KT), allows one to operate on finite strings of arbitrary length. It is of high demand in digital image processing and computer vision but features a prohibitive runtime. Here, we report a one-step computation of a fractional quantum KT. The quantum *d*-nary (qudit) architecture we use comprises only one gate and offers processing time independent of the input size. The gate may use a multiphoton Hong-Ou-Mandel effect. Existing quantum technologies may scale it up toward diverse applications.

## Teaser

We found that quantum interference allows processing large sets of data faster and more accurately than computer algorithms.

## INTRODUCTION

Science, medicine, and engineering demand efficient information processing. It is a long-standing goal to use quantum mechanics to substantially improve these computations (*1*). The processing routinely involves examining data as a function of complementary variables, e.g., time and frequency. This is done by the Fourier transform (FT) approximations, which accurately compute inputs of 2*^n^* samples in *O*(*n* 2*^n^*) steps (*2*). In the quantum domain, an analogous process exists, namely an FT of quantum amplitudes (*3*), which requires exponentially fewer *O*(*n* log *n*) quantum gates. Here, we report a quantum fractional Kravchuk-Fourier transform (KT), a related process suited to finite string processing (*4*). Unlike previous demonstrations (*5*, *6*), our architecture involves only one gate, resulting in constant-time processing of quantum information. The gate exploits a generalized Hong-Ou-Mandel (HOM) effect (*7*), the basis for quantum-photonic information applications (*8*). We perform a proof-of-concept experiment by the creation of large photon number states, interfering them on a beam splitter (BS) and using photon-counting detection. The discrete FT (DFT) is an efficient approximation to the FT. The signal (*x*_0_, *x*_1_,…, *x_S_*) is taken to be samples of one period of a continuous function and is turned into a new sequence (*X*_0_, *X*_1_, …, *X_S_*) where

Xk=1S+1∑l=0Se−i2πklS+1·xl,k=0,…,S (1)

The DFT does not, however, reproduce all essential features of the FT. In some cases, a transform that is a fractional power of the FT, the α-fractional FT where 0 ≤ α ≤ 1, yields advantages (*9*). For α = 0, this transform is the identity, while for α = 1, this is the FT. If $\alpha =\frac{1}{n}$, where *n =* 2, 3, 4,…, then a composition of *n* α-fractional FTs amounts to the FT. This intuitive property does not hold true for the α-fractional DFT (Supplementary Materials), which generalizes the DFT, but for α = 1, it reduces to Eq. 1. This is because the α-fractional FT can be seen as a circular rotation of the signal in the time-frequency plane by angle $\frac{\pi \alpha }{2}$, while the α-fractional DFT is an elliptical rotation in this plane, which requires additional computation steps to properly approximate the α-fractional FT (*9*).

The DFT is powerful because of the fast FT (FFT) algorithm (*2*). Using an FFT lowers the number of operations from *O*(2^2*n*^) to *O*(*n* 2*^n^*), which nevertheless remains a bottleneck in signal processing (*10*). The FFT uses a “divide and conquer” method to recursively split Eq. 1 into 2*^n^* sums, which can be processed quickly, and therefore is applicable to signals of period 2*^n^*. Notably, the minimal number of operations required to implement the DFT is unknown (*11*). The quantum FT, the cornerstone of quantum algorithms (*12*, *13*), enables implementation of the DFT on quantum amplitudes with *O*(*n* log *n*) operations by processing *n* qubits (*n* quantum bits encode 2*^n^* amplitudes) (*14*).

In many applications, e.g., bioimaging, the signals are typically not periodic and are random in length. For these cases, the KT is a useful alternative to the FFT because it can be applied to finite signal processing (*15*, *16*). The KT computes orthogonal moments corresponding to the Kravchuk polynomials, which are discrete and orthogonal with respect to a binomial distribution in the data space (*4*). By varying a parameter of the binomial distribution, one is able to set the fractionality α of the KT (Supplementary Materials). This feature allows the exploration of a specific region of interest of an image. To illustrate the action of a KT, the numerical study in fig. S5 in the Supplementary Materials demonstrates the advantages of KT over FFT in reconstructing test images.

The KT’s computational time is equal to the DFT’s runtime (*17*) (Supplementary Materials), and implementations with a lower number of operations are of high demand. Recently, quantum KTs (QKTs) have been realized in waveguides with two photons, but they are difficult to scale up and their fractionality is fixed by waveguide length (*5*, *6*).

The α-fractional KT uses the weighted Kravchuk polynomials $\phi_{k}^{\left(p\right)}(q,S)$ (*4*), which are real-valued and correspond to wave functions of finite harmonic oscillators

Xk=∑l=0Se−iπα2S2eiπ2(l−k)ϕk(p)(l−Sp,S)·xl,k=0,…,S (2)

where $p={\text{sin}}^{2}(\frac{\pi \alpha }{4})$. Unlike plane waves, $e^{-i2\pi \frac{\mathrm{kl}}{S+1}}$, the polynomials are defined and orthogonal on a set of *S* + 1 points. This enables one to transform the signal as a finite sequence rather than as an infinite periodic one. In the limit of *S* → ∞, $\phi_{k}^{\left(p\right)}(q,S)$ tend to eigenfunctions of quantum harmonic oscillators and the α-fractional KT reproduces the α-fractional FT. Equation 2 can be viewed in terms of overlaps of two spin-$\frac{S}{2}$ states, in which they are prepared as eigenstates of *S*_3_ and one undergoes a rotation by angle $\frac{\pi \alpha }{2}$ generated by *S*_1_, $e^{i\frac{\pi }{2}(l-k)}\phi_{k}^{\left(p\right)}(l-\mathrm{Sp},S)=\langle \frac{S}{2};\frac{S}{2}-k|e^{i\frac{\pi \alpha }{2}S_{1}}|\frac{S}{2};\frac{S}{2}-l\rangle$.

## RESULTS

Here, we demonstrate a single-step QKT with tunable fractionality using quantum effects based on multiparticle bosonic interference resulting from an exchange interaction. To this end, we interfere photon number states (light pulses with definite particle number) on a BS with an adjustable splitting ratio. This leads to a multiparticle HOM effect (*18*), which we observe using states with up to five photons. This QKT implementation enables constant-time quantum information processing for quantum *d*-nary (qudit) data encoding, which is set by the total number of interfering particles *S*, allowing up to *d* = *S* + 1 signal samples.

**Fig. 1:**
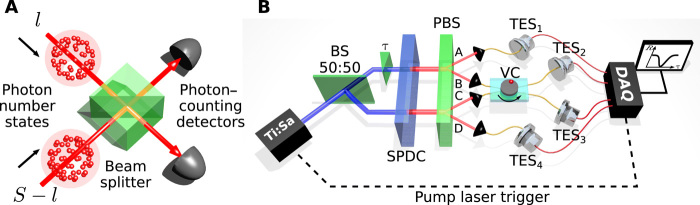
Photonic implementation of a fractional QKT. (**A**) HOM interference of photon number states on a variable BS, followed by two photon-counting detectors, (**B**) Setup: Ti:Sa, titanium-sapphire laser pump (blue); BS, 50:50 BS; τ, optical phase delay; SPDC, periodically poled potassium titanyl phosphate nonlinear spontaneous parametric down-conversion waveguide chip that produces photon number–correlated states (red); VC, variable coupler; DAQ, data acquisition unit.

Photon number (Fock) states $|l\rangle =\frac{{\left(a^{†}\right)}^{l}}{\sqrt{l}}|0\rangle$ and $|S-l\rangle =\frac{{\left(b^{†}\right)}^{S-l}}{\sqrt{S-l}}|0\rangle$ impinging on a BS exhibit a generalized HOM effect (Fig. 1A). A BS interaction between two such inputs described by annihilation operators *a* and *b* is $U_{\text{BS}}=\text{exp}\{\frac{\theta }{2}(a^{†}be^{-i\varphi }-ab^{†}e^{i\varphi })\}$, where $r={\text{sin}}^{2}\frac{\theta }{2}$ is the BS reflectivity (defined as the probability of reflection of a single photon) and φ is the phase difference between the reflected and transmitted fields (*19*). Since φ does not influence our experiments, we assume that $\varphi =\frac{\pi }{2}$ for convenience. If the BS is balanced (*r* = 0.5), then two photons at the input ports will leave through the same exit port. This is known as photon bunching (*7*). Similar effects hold for multiphoton number states (*18*). This is reflected in the probability amplitudes of detecting ∣*k*〉 and ∣*S* − *k*〉 behind the BS, $A_{S}^{\left(r\right)}(k,l)=e^{-i\theta \frac{S}{2}}\langle k,S-k|U_{\text{BS}}|l,S-l\rangle$. This is important for implementing the KT, since $A_{S}^{\left(r\right)}(k,l)=e^{-i\theta \frac{S}{2}}e^{i\frac{\pi }{2}(l-k)}\cdot \phi_{k}^{\left(r\right)}(l-\mathrm{Sr},S)$; thus, if we send a quantum state $|\Psi \rangle =\sum_{l=0}^{S}x_{l}|l,S-l\rangle$ into the BS, then the probability of measuring *k* and *S* − *k* photons behind is the absolute square of a fractional QKT of the input probability amplitudes, ${|X_{k}|}^{2}={|\sum_{l=0}^{S}A_{S}^{\left(r\right)}(k,l)\cdot x_{l}|}^{2}$ (compare Eq. 2). The reflectivity *r* determines the QKT fractionality, $\alpha =\frac{2\theta }{\pi }=\frac{4}{\pi }\text{arcsin}\sqrt{r}$. Since two-mode optical interference can be achieved in a single step, regardless of the number of photons involved, this process implements a constant time QKT. For full derivations, see the Supplementary Materials.

**Fig. 2:**
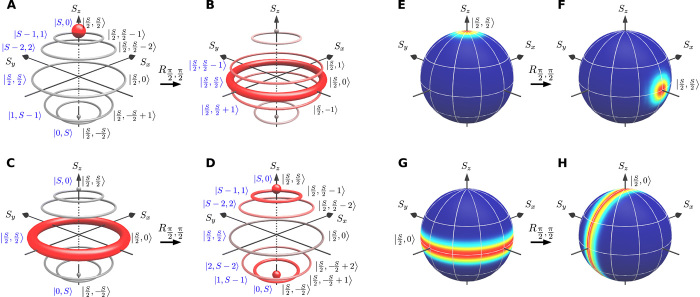
HOM interference and QKT on a Bloch sphere. (**A** to **D**) Two-mode Fock states (blue) correspond to Dicke states (black)—the basis of spin-$\frac{S}{2}$ states. HOM interference turns Dicke states into a superposition of them. This coincides with a rotation *R*_θ,ϕ_ in the Dicke state basis. The two most distinct cases are shown: the rotation $R_{\frac{\pi }{2},\frac{\pi }{2}}$ of the pole $|\frac{S}{2};\frac{S}{2}\rangle$ and of the great circle state $|\frac{S}{2};0\rangle$. (**E** to **H**) Q-function representation of (A to D). HOM interference implements a rotation on the Bloch sphere by $\theta =\frac{\pi }{2}$ around *S_x_* of input *S_z_*-eigenbasis Dicke states and thus the full QKT (compare Eq. 2). The sequence (*x*_0_, *x*_1_,…, *x_S_*) is (1, 0, 0, …, 0) in (A) and (0, …, 1, …, 0) in (C). The QKT transfers the input—a position eigenstate—into the same state but in *S_y_* basis—a momentum eigenstate.

A deeper understanding of the result may be gained from the Schwinger representation of the spin algebra (Supplementary Materials), which links multiphoton interference to spin systems and allows the quantum states to be visualized on a Bloch sphere. In this picture, a total of *S* photons correspond to a spin-$\frac{S}{2}$ system. The Hamiltonian generating *U*_BS_ = exp { −*i*θ*H*_BS_} corresponds to an *S_x_* operator for a spin-$\frac{S}{2}$. The two-mode Fock state ∣*l*, *S* − *l*〉 corresponds to an $S_{z}=\frac{S}{2}-l$ eigenstate, known as a Dicke state. Hence, HOM interference may be considered a rotation *R*_θ, φ_ = exp { −*i*θ*S_x_*} of *S_z_* around the *S_x_* axis on the sphere. It transfers the eigenstate $|\frac{S}{2};\frac{S}{2}-l\rangle$ to a superposition of Dicke states (Fig 2, A to D). The Q-function in Fig. 2 (E to H) shows that the initial and final states are eigenstates of two complementary observables, *S_z_* and *S_y_*, respectively. Thus, one may identify the former with a position, and the latter with a momentum eigenstate.

We depicted experimental setup for multiphoton HOM interference in Fig. 1B. Two pulsed spontaneous parametric down-conversion (SPDC) sources each generated two-mode photon number–correlated states (Supplementary Materials). The signal and idler were separated with a polarization BS (PBS) into four spatial modes. The modes *A* and *D* were used for heralding and creation of Fock states ∣*l*〉 in *B* and ∣*S* − *l*〉 in *C*, which interfered in a variable ratio fiber coupler (the BS). An optical phase delay τ in one of the pump beams ensured optimal temporal overlap at interference. Photon number–resolved measurements were achieved using transition edge sensors (TESs) that we previously estimated to achieve over 90% efficiency (*20*).

**Fig. 3:**
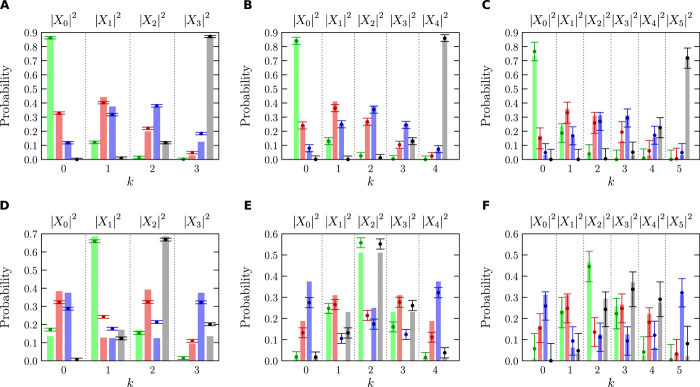
Photon number statistics resulting from Fock state |*l*, *S − l*〉 interference. The probabilities of detecting ∣*k*〉 and ∣*S* − *k*〉 photons behind the BS for input (**A**) ∣0,3〉, (**B**) ∣0,4〉, (**C**) ∣0,5〉, (**D**) ∣1,2〉, (**E**) ∣2,2〉, and (**F**) ∣2,3〉. The BS reflectivities are *r* = 0.05 (green), 0.2 (red), 0.5 (blue), and 0.95 (gray). Vertical bars represent theoretical values for an ideal system, while dots are values determined in experiment. The states in (A) to (C) encode sequences (*x*_0_ = 1, *x*_1_ = 0, …, *x_S_* = 0), and states in (D) to (F) encode (0, 1, 0, 0), (0, 0, 1, 0, 0), and (0, 0, 1, 0, 0, 0), respectively. The measured probabilities set their QKTs (∣*X*_0_∣^2^, ∣*X*_1_∣^2^, …, ∣*X_S_*∣^2^), ${|X_{k}|}^{2}={|\sum_{l=0}^{S}A_{S}^{\left(r\right)}(k,l)\cdot x_{l}|}^{2}$ of fractionality α = 0.28 (green), 0.60 (red), 1.00 (blue), and 1.72 (gray).

We interfered the vacuum ∣0〉 (*l* = 0) with multiphoton Fock states ∣*S*〉 (*S* − *l* = *S*) on a coupler with splitting ratios *r* = 0.05 (green), 0.2 (red), 0.5 (blue), and 0.95 (gray) and measured photon number statistics. They are depicted in Fig. 3 (A to C) for *S* = 3, 4, 5. The input states encode sequences (*x*_0_ = 1, *x*_1_ = 0,…, *x_S_* = 0), while the measured probabilities set their QKTs to (∣*X*_0_∣^2^, ∣*X*_1_∣^2^, …, ∣*X_S_*∣^2^), where ${|X_{k}|}^{2}={|A_{S}^{\left(r\right)}(k,0)|}^{2}$. The reflectivities used correspond to fractionalities α = 0.28, 0.60, 1.00, and 1.72. Errors were estimated as a square root inverse of the number of measurements (Supplementary Materials). The second-order interferometric visibility reached values between 71.4 and 98.6% for *S* = 5 (Supplementary Materials).

For the same values of *r*, we measured photon number distribution resulting from interference of ∣1,2〉, ∣2,2〉, and ∣2,3〉. They are shown in Fig. 3 (D to F). The inputs encode (0, 1, 0, 0), (0, 0, 1, 0, 0), and (0, 0, 1, 0, 0, 0), while ${|X_{k}|}^{2}={|A_{3}^{\left(r\right)}(k,1)|}^{2}$, ${|A_{4}^{\left(r\right)}(k,2)|}^{2}$, and ${|A_{5}^{\left(r\right)}(k,2)|}^{2}$. The visibility was between 54.8 and 99.5% (*S* = 5) (Supplementary Materials). Figure 3 shows that the theoretical values computed for an ideal system (the bars) match the experimental results (the dots) well.

## DISCUSSION

Realization of the fractional QKT with qudit systems opens a previously unidentified prospect for transformation of large data sequences in *O*(1) time. This is not possible with the implementations based on waveguides. Both cases are examples of a nonuniversal quantum computer optimized for one task, which is the basis for a variety of important applications (*15*). The photonic proof of concept is currently limited by the range of input states that can be prepared. However, deterministic creation of an arbitrary superposition of Fock states has been demonstrated for trapped ions and superconducting resonators (*21*). Since a BS sees orthogonal spectral or polarization modes independently, one can extend the transform to higher dimensions (*22*, *23*). We note that the QKT could also be implemented on existing quantum annealing processors (*24*), which operate on a chain of interacting spin-$\frac{1}{2}$ systems (Supplementary Materials), and using HOM interference of fermions with a symmetric wave function of the interfering degrees of freedom.

Large-scale realizations of the QKT may use an increasing number of measurements as an error minimization strategy (Supplementary Materials). This is akin to the common approach in classical data processing where the accuracy can be improved by an enhanced precision of numeric data types and number of iterations, without altering the proper algorithm. Errors resulting from losses in the system, e.g., at the BS, are easily corrected by applying a post-selection scheme and filtering out the cases when the total number of photons behind the BS is lower than in the input state.

*O*(1) computation of the fractional FT for continuous variable systems can be implemented with a shallow system, realizing a phase shift operation (*25*). This is a quantum counterpart of the operation of a single focusing lens in classical optics, which produces the fractional FT of the image placed at its focal length (*9*). The QKT operates on discrete variables, but when the sampling rate and the sequence length of input data increase, the α-fractional QKT tends to the α-fractional FT (*4*). This relation is also reflected by the fact that symmetric Kravchuk functions $\phi_{k}^{\left(p=1/2\right)}$ (eigenfunctions of QKT) tend to Hermite-Gauss polynomials (eigenfunctions of the FT) in this limit.

Our result, along with the fact that qudit-based algorithms exhibit much lower number of operations than qubit-based ones (*26*), motivates the further development of highly controllable quantum harmonic oscillator platforms with implications for quantum signal processing in a whole range of applications. Provided efficient input state preparation and detection of larger Fock states, the *O*(1) QKT demonstrated here, in principle, may find practical applications in imaging of unprecedented quality, fostering early diagnostics and advances in neuroscience (*27*).

## MATERIALS AND METHODS

A light pulse from a Ti:Sapphire laser at 775 nm [full width at half maximum (FWHM), 2 nm; repetition rate, 75 kHz] pumped collinear type-II phase-matched 8-mm-long SPDC waveguides written in a periodically poled potassium titanyl phosphate crystal sample. They generate two independent photon number–correlated states, the two-mode squeezed vacua $|\Psi \rangle =\sum_{n=0}^{\infty }\lambda_{n}|n,n\rangle$, where $\lambda_{n}=\frac{{\text{tanh}}^{n}g}{\text{cosh}g}$ is a probability amplitude for creation of a pair of *n* photons and *g* is the parametric gain. The average photon number in the signal and idler mode equals $\langle \hat{n}\rangle ={\text{sinh}}^{2}g$. For small *g*, cosh *g* ≈ 1, and thus, $\lambda_{n}^{2}\approx {\text{sinh}}^{2n}g={\langle \hat{n}\rangle }^{n}$. In the experiment, the average photon number was $\langle \hat{n}\rangle \approx 0.2$. This value is sufficient to ensure the emission of multiphoton pairs but, at the same time, to diminish the interferometric visibility of two-photon events. In both output states, the signal and idler pulses were split with a PBS to four spatial modes *A* to *D*. Subsequently, they were filtered by bandpass filters with an FWHM of 3 nm angle-tuned to the central wavelength of their respective spectra to reduce the broadband background typically generated in dielectric nonlinear waveguides (*28*). The pump beam was discarded with an edge filter. The modes *A* and *D* were used for heralding and conditional creation of Fock states in modes *B* and *C*, which interfered in a variable ratio polarization-maintaining (PM) fiber coupler. The coupling ratio can be set in the range of 0 to 100% with an error of ±1.5%. The heralding signal modes (H-pol.) were centered at 1554 nm, while the interfering idler modes (V-pol.) were at 1546 nm. We used TESs running at 70 mK, which allowed for photon number–resolved measurements in all modes (*29*). Their voltage output was captured with an analog to digital converter (ADC) card.

Before demonstrating the HOM interference, we characterized the setup. A high–photon number resolution and single-mode input states are pivotal for this experiment. The resolution of TES detectors (the confidence that the detector gives a correct information about the number of photons) was previously confirmed to exceed 95% (*20*). The depth of the HOM dip of 85.9 ± 0.3% for a two-photon interference indicated an effective Schmidt-mode number *K* = 1.16. For the measured 4-tuples of photon numbers, losses were computed by assuming perfect setup components, each followed by a BS with a reflection coefficient introducing the loss. We estimated that the total transmission in each mode to be approximately 50%. For the details, see the Supplementary Materials.

Measurements for individual settings of the splitting ratio were taken over approximately 400 s, giving 10^9^ data samples for each *r* ranging from 0 to 1 with a step of approximately 3%. Small error bars for low photon numbers and larger bars for the higher ones result from keeping the pump power fixed and near-single modeness of the interfering beams.

## Supplementary Material

http://advances.sciencemag.org/cgi/content/full/5/7/eaau9674/DC1

Download PDF

Quantum interference enables constant-time quantum information procesing

## SUPPLEMENTARY MATERIALS

Supplementary material for this article is available at http://advances.sciencemag.org/cgi/content/full/5/7/eaau9674/DC1 Fig. S1. Symmetric Kravchuk polynomials *k_n_*^(1/2)^(*x*, *N*) and functions ϕ*_n_*^(1/2)^(*x*, *N*). Fig. S2. Basis states for a 16-point KT. Fig. S3. Basis states for a 16-point discrete FT. Fig. S4. KT versus DFT. Fig. S5. Example of FFT and KT image processing. Fig. S6. HOM dip. Fig. S7. Photon number statistics resulting from Fock state ∣*l*, *S* − *l*〉 interference. Table S1. Second-order interferometric visibilities in HOM interference. References (*30*–*40*)
