# Supplementary material for: Quantum interference enables constant-time quantum information processing
Source: Sci Adv. 2019 Jul 19;5(7):eaau9674. doi: 10.1126/sciadv.aau9674 (PMC6641944; doi:10.1126/sciadv.aau9674)
Supplement: http://advances.sciencemag.org/cgi/content/full/5/7/eaau9674/DC1 [file supp_5_7_eaau9674__index.html]

Science Advances | Science AdvancesAAASSearchScience AdvancesMenu

## Supplementary Materials

**This PDF file includes:**

- Fig. S1. Symmetric Kravchuk polynomials *kn*(1/2)(*x*, *N*) and functions ϕ*n*(1/2)(*x*, *N*).
- Fig. S2. Basis states for a 16-point KT.
- Fig. S3. Basis states for a 16-point discrete FT.
- Fig. S4. KT versus DFT.
- Fig. S5. Example of FFT and KT image processing.
- Fig. S6. HOM dip.
- Fig. S7. Photon number statistics resulting from Fock state ∣*l*, *S* − *l*〉 interference.
- Table S1. Second-order interferometric visibilities in HOM interference.
- References (*30*–*40*)

Download PDF

**Files in this Data Supplement:**

- Adobe PDF - aau9674\_SM.pdf
