# Supplementary material for: Quantum interference enables constant-time quantum information processing
Source: Sci Adv. 2019 Jul 19;5(7):eaau9674. doi: 10.1126/sciadv.aau9674 (PMC6641944; doi:10.1126/sciadv.aau9674)
Supplement: Download PDF [file aau9674_SM.pdf]

Science Advances
AAAS

advances.sciencemag.org/cgi/content/full/5/7/eaau9674/DC1

Supplementary Materials for

Quantum interference enables constant-time quantum information processing

M. Stobińska*, A. Buraczewski, M. Moore, W. R. Clements, J. J. Renema, S. W. Nam, T. Gerrits, A. Lita, W. S. Kolthammer, A. Eckstein, I. A. Walmsley

*Corresponding author. Email: magdalena.stobinska@gmail.com

Published 19 July 2019, *Sci. Adv.* **5**, eaau9674 (2019)
DOI: 10.1126/sciadv.aau9674

This PDF file includes:

Fig. S1. Symmetric Kravchuk polynomials $k_n^{(1/2)}(x, N)$ and functions $\phi_n^{(1/2)}(x, N)$.
Fig. S2. Basis states for a 16-point KT.
Fig. S3. Basis states for a 16-point discrete FT.
Fig. S4. KT versus DFT.
Fig. S5. Example of FFT and KT image processing.
Fig. S6. HOM dip.
Fig. S7. Photon number statistics resulting from Fock state $|l, S-l\rangle$ interference.
Table S1. Second-order interferometric visibilities in HOM interference.
References (*30–40*)

Supplementary Materials

1 Mathematical foundations for the Kravchuk transform

1.1 Kravchuk matrices and polynomials

Kravchuk polynomials are related to Kravchuk matrices that are generated by a binomial expressions of the form $(1+x)^{N-j}(1-x)^j$, with positive integer N and $j = 0, \ldots, N$. Such an expression expands to a set of $N+1$ polynomials $a_{0,j} + a_{1,j}x + a_{2,j}x^2 + \ldots + a_{i,j}x^i + \ldots + a_{N,j}x^N$ Their coefficients can be used to construct a square $(N+1) \times (N+1)$ matrix $\mathbf{K}^{(N)}$ with an entry in ith row and jth column given by $\mathbf{K}^{(N)}_{i,j} = a_{i,j}$. Thus

$$(1+x)^{N-j}(1-x)^j = \sum_{i=0}^{N} x^i \, \mathbf{K}^{(N)}_{i,j} \tag{S1}$$

As an example, let us examine the expression $(1+x)^{N-j}(1-x)^j$ for $N = 3$ and $j = 0, \ldots, 3$. Its all possible expansions read

$$\begin{aligned}
j = 0: &\qquad (1+x)^3 = 1 + 3x + 3x^2 + x^3 \\
j = 1: &\qquad (1+x)^2(1-x) = 1 + x - x^2 - x^3 \\
j = 2: &\qquad (1+x)(1-x)^2 = 1 - x - x^2 + x^3 \\
j = 3: &\qquad (1-x)^3 = 1 - 3x + 3x^2 - x^3
\end{aligned}$$

and thus, the corresponding Kravchuk matrix is as follows

$$\mathbf{K}^{(3)} = \begin{pmatrix} 1 & 1 & 1 & 1 \\ 3 & 1 & -1 & -3 \\ 3 & -1 & -1 & 3 \\ 1 & -1 & 1 & -1 \end{pmatrix}$$

Kravchuk matrices possess many interesting properties (*30*). The top row contains only 1's, while the bottom one $(-1)^j$. The first column is expressed by binomial coefficients $\binom{N}{i}$ and the last one by $(-1)^i\binom{N}{i}$. The matrix is also characterized by a four-fold symmetry $|\mathbf{K}^{(N)}_{i,j}| = |\mathbf{K}^{(N)}_{N-i,j}| = |\mathbf{K}^{(N)}_{i,N-j}| = |\mathbf{K}^{(N)}_{N-i,N-j}|$. Finally, $\left(\mathbf{K}^{(N)}\right)^2 = 2^N\,\mathbf{I}$, where $\mathbf{I}$ is the identity matrix. The latter property is important as it underpins the existence of the Kravchuk transform. Kravchuk matrices are considered to be generalized Pascal triangles, while the columns of $\mathbf{K}^{(N)}$ are called generalized binomial coefficients. The entries of $\mathbf{K}^{(N)}$ can be computed using the following formula

$$\mathbf{K}^{(N)}_{i,j} = \sum_{k=0}^{i}(-1)^k\binom{j}{k}\binom{N-j}{i-k} = k_i^{(1/2)}(j,N) \tag{S2}$$

where $k_n^{(1/2)}(x,N)$ denotes n-th symmetric Kravchuk polynomial of variable x and order N.

Kravchuk polynomials are defined by the rows of $\mathbf{K}^{(N)}$. Their domain is $x = 0,\ldots,N$. They fulfill the following orthogonality relation

$$\frac{1}{2^N}\sum_{j=0}^{N}\binom{N}{j}k_n^{(1/2)}(j,N)\,k_m^{(1/2)}(j,N) = \frac{1}{2^{2n}}\binom{N}{n}\delta_{n,m} \tag{S3}$$

where n and m are integers and $\delta_{m,n}$ is the Kronecker delta equal 1 where $n = m$ and 0 otherwise.

In case of an unsymmetric binomial expression, the Kravchuk matrix can be generated like this

$$(1+(p-1)x)^{N-j}(1-x)^j = \sum_{i=0}^{N}x^i\mathbf{K}^{(p,N)}_{i,j} = \sum_{i=0}^{N}x^i k_i^{(p)}(j,N) \tag{S4}$$

where $0 \leq p \leq 1$ and the Kravchuk polynomials

$$k_i^{(p)}(j,N) = \sum_{k=0}^{i} (-1)^k (p-1)^{i-k} \binom{j}{k} \binom{N-j}{i-k} \tag{S5}$$

fulfill the following orthogonality relation

$$\sum_{j=0}^{N} \binom{N}{j} (p-1)^j k_n^{(p)}(j,N)\, k_m^{(p)}(j,N) = p^N (p-1)^n \binom{N}{n} \delta_{n,m} \tag{S6}$$

Kravchuk polynomials belong to the family of special functions and can also be expressed in terms of a Gauss hypergeometric function ${}_2F_1[a,b;c;z]$ (see Section 4)

$$k_n^{(p)}(x,N) = (-1)^n \binom{N}{n} p^n \, {}_2F_1\left[-n,-x;-N;\tfrac{1}{p}\right] = (-1)^n \binom{N}{n} p^n \sum_{i=0}^{n} \binom{n}{i} (-1)^i \frac{(-x)_i}{(-N)_i} \frac{1}{i!\,p^i} \tag{S7}$$

where $(a)_i = a \cdot (a+1) \cdot \ldots \cdot (a+i-1)$ is the Pochhammer symbol. From Eq. S7 it is clear that $k_n^{(p)}(x,N)$ are polynomials of degree n and that the parameter n and variable x can be exchanged in the following way

$$(-1)^x \binom{N}{x} p^x k_n^{(p)}(x,N) = (-1)^n \binom{N}{n} p^n k_x^{(p)}(n,N) \tag{S8}$$

Kravchuk polynomials can be regarded as a discrete and finite counterpart of Hermite polynomials $H_n(x)$ (*4*)

$$\lim_{N\to\infty} \left(\frac{2}{Np(1-p)}\right)^{n/2} n!\, k_n^{(p)}\left(Np + x\sqrt{2Np(1-p)}, N\right) = H_n(x) \tag{S9}$$

1.2 Kravchuk functions

Kravchuk functions $\phi_n^{(p)}$ are Kravchuk polynomials that are normalized and centered at the maximum of a binomial distribution $\binom{N}{x} p^x (1-p)^{N-x}$

$$\phi_n^{(p)}(x-Np,N) = \sqrt{\frac{n!(N-n)!}{x!(N-x)!}} \sqrt{p^{x-n}(1-p)^{N-n-x}} k_n^{(p)}(x,N) \tag{S10}$$

Their domain is $x = -Np, -Np+1, \ldots, -Np+N$. They are orthonormal

$$\sum_{i=0}^{N} \phi_n^{(p)}(i-Np,N)\, \phi_m^{(p)}(i-Np,N) = \delta_{n,m} \tag{S11}$$

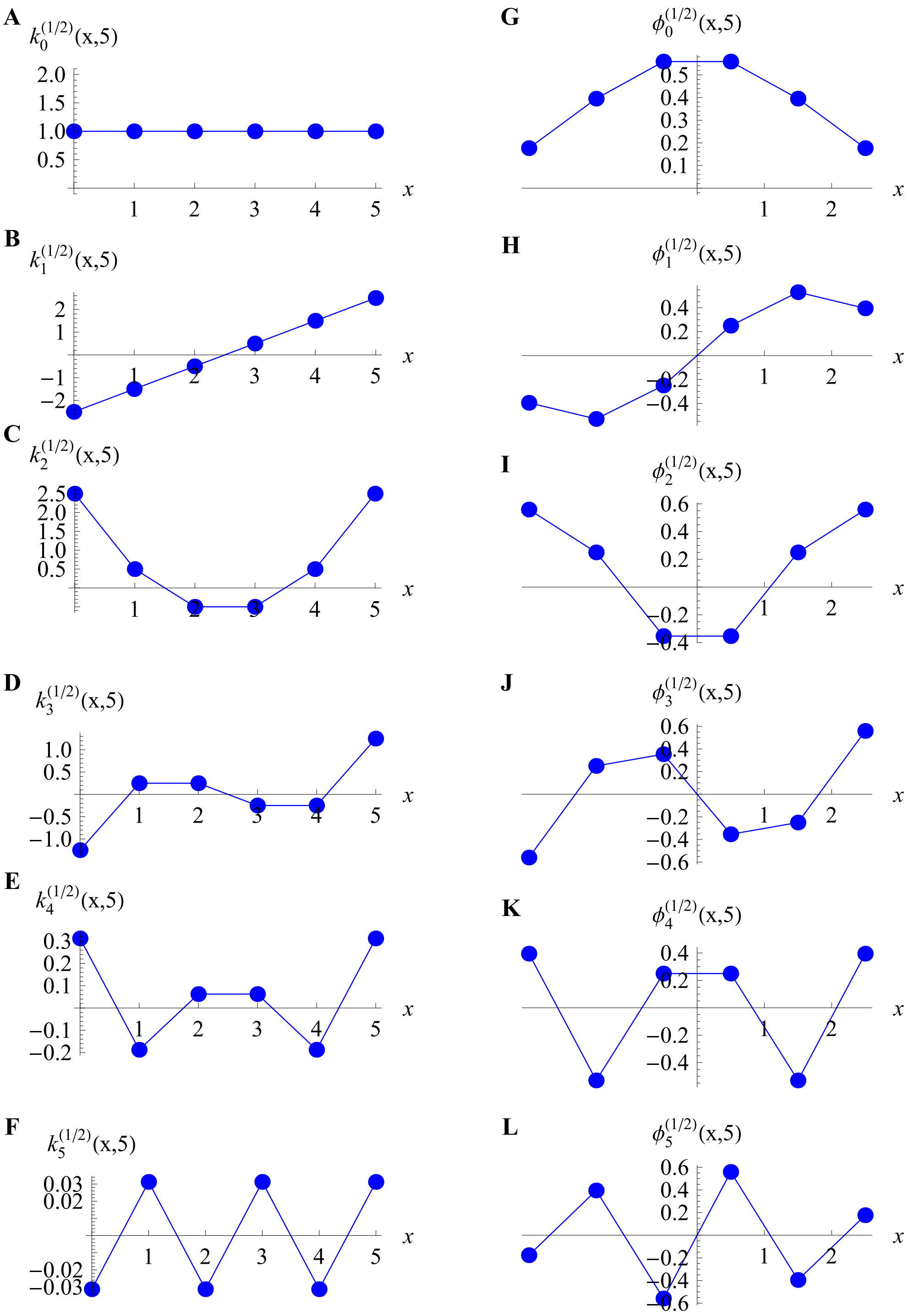

Fig. S1. Symmetric Kravchuk polynomials $k_n^{(1/2)}(x, N)$ and functions $\phi_n^{(1/2)}(x, N)$. The plots (**A**)-(**F**) show Kravchuk polynomials, while (**G**)-(**L**) – Kravchuk functions. Computations are performed for $N = 5$.

and the variables n and i can be used interchangeably

$$\phi_n^{(p)}(i - Np, N) = \phi_i^{(p)}(n - Np, N) \tag{S12}$$

Interestingly, Kravchuk functions are solutions of finite oscillator wave equation

$$\mathbf{H}^{(N)}(x)\,\phi_n^{(1/2)}(x, N) = (n + \tfrac{1}{2})\,\phi_n^{(1/2)}(x, N), \qquad n = 0, \ldots, N \tag{S13}$$

where $\mathbf{H}^{(N)}$ is a finite-difference operator identified as a discrete Hamiltonian of this system. In the limit of $N \to \infty$ Kravchuk functions tend to the harmonic oscillator wave functions $\psi_n(x)$ (Hermite–Gauss polynomials)

$$\lim_{N\to\infty} (N/2)^{1/4} \phi_n^{(1/2)} \left(x\sqrt{N/2}, N\right) = \psi_n(x) \tag{S14}$$

where

$$\psi_n(x) = \frac{1}{\sqrt{\sqrt{\pi}\,2^n\,n!}}\,H_n(x)\,e^{-x^2/2} \tag{S15}$$

Fig. S1 shows Kravchuk polynomials and Kravchuk functions for $N = 5$.

1.3 Kravchuk transform

The Kravchuk transform (KT) is defined by means of Kravchuk functions. KT converts an input sequence $(x_0, x_1, \ldots, x_S)$ into a new string $(X_0, X_1, \ldots, X_S)$ in the following way (cf. the main text)

$$X_k = \sum_{l=0}^{S} e^{-i\frac{\pi\alpha}{2}\frac{S}{2}} e^{i\frac{\pi}{2}(l-k)}\,\phi_k^{(p)}(l - Sp, S) \cdot x_l, \qquad k = 0, \ldots, S \tag{S16}$$

where α is the fractionality of the transform and $p = \sin^2(\pi\alpha/4)$. Effectively, it decomposes the input string in the basis of Kravchuk functions. The KT can also be seen as multiplication of the input vector by a rescaled Kravchuk matrix $\mathbf{K}^{(N)}$ with additional phase terms.

As an illustration, Fig. S2 presents the full set of basis states of a 16-point Kravchuk transform ($S = 15$ and $k = 0, \ldots, 15$).

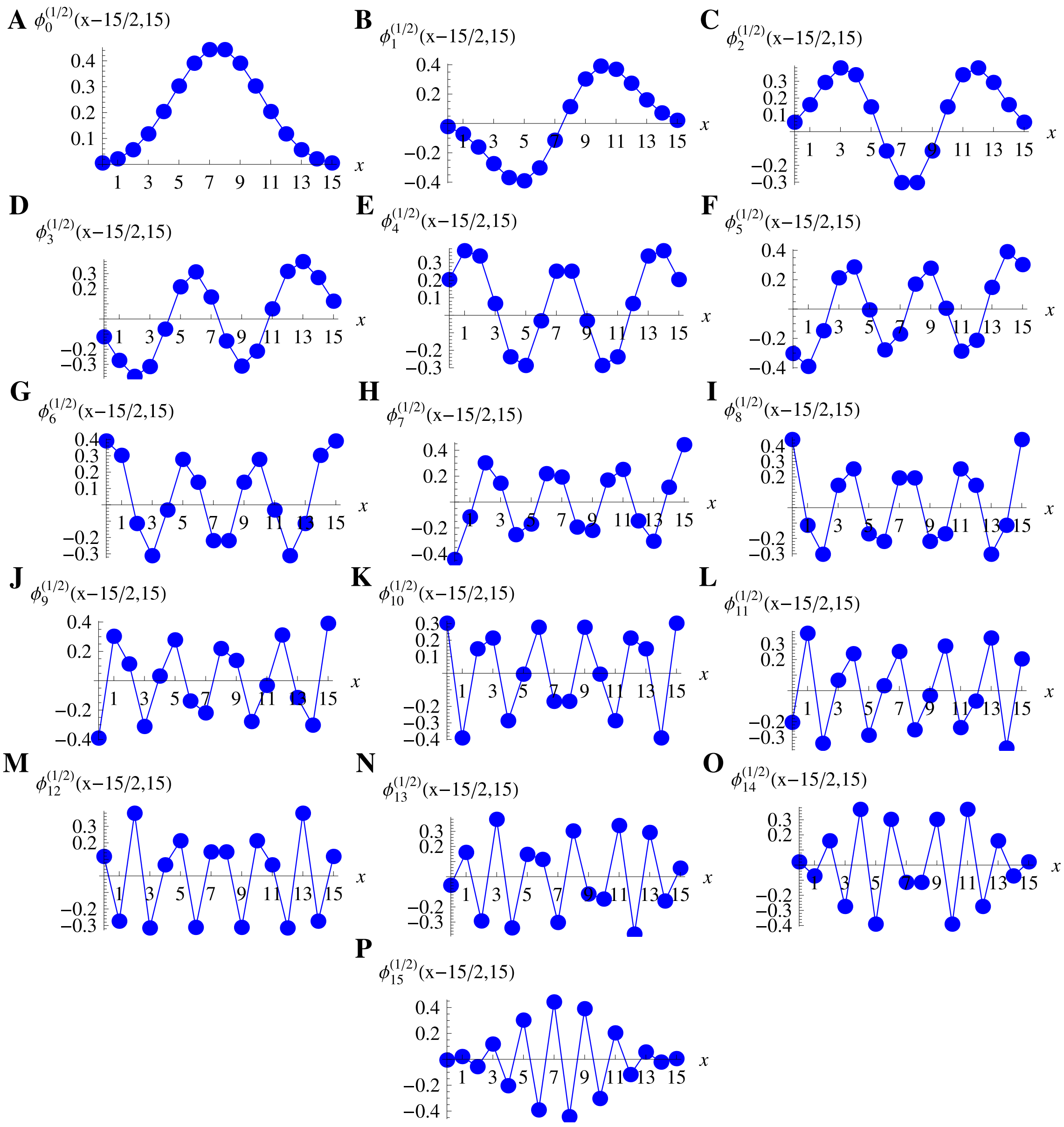

Fig. S2. Basis states for a 16-point KT. Plots (**A**)-(**P**) depict orthonormal basis states $\phi_k^{(1/2)}(x - S/2, S)$ for $S = 15$ and $k = 0, \ldots, S$, respectively.

The parameter α modifies the Kravchuk transform (Eq. S16) in the following way

1. for $\alpha = 1$, it is a forward transform,
2. for $\alpha = 2$, it is an inverse (backward) transform, allowing one to compute the original sequence (x_l) from (X_k) using the same algorithm or a physical system,
3. for $\alpha = 3$, the transform negates the input, $X_k = -x_k$,
4. for $\alpha = 0$ or for $\alpha = 4$, the transform is an identity operation, $X_k = x_k$.

Similar properties are observed for the (integral) Fourier transform.

In addition, the Fourier transform and the KT can be seen as circular rotations of the data in the time-frequency space by an angle $\theta = \pi\alpha/2$. This rotation is also well-defined for $0 < \alpha < 1$, leading to an α-fractional transforms. The KT is additive with respect to α, i.e. application of two consecutive transforms parameterized by α_1 and α_2 results in a α-fractional KT with $\alpha = \alpha_1 + \alpha_2$.

1.4 Discrete Fourier transform

The discrete Fourier transform (DFT) as well as the Fast Fourier Transform (FFT) algorithm decompose input data string in the basis of plane waves

$$X_k = \frac{1}{\sqrt{S+1}} \sum_{l=0}^{S} e^{-i2\pi\frac{kl}{S+1}} \cdot x_l \tag{S17}$$

Fig. S3 depicts the full set of basis states of a 16-point discrete Fourier transform ($S = 15$ and $k = 0, \ldots, 15$).

Since plane waves $e^{-i2\pi\frac{kl}{S+1}}$ are periodic and their domain consist of negative and positive integers and zero, the discrete Fourier transform defined in Eq. S17 correctly decomposes only samples of periodic data. Since the Fourier transform of a discrete periodic signal with period of length $S+1$ results also in a discrete periodic output of the same length, it is enough to process one period of the input string, $(x_0, \ldots, x_S)$, and store one period of the output sequence $(X_0, \ldots X_S)$. However, one should remember that effectively the input sequence is seen by this algorithm as infinite

$$\ldots, x_0, x_1, \ldots, x_S, x_0, x_1, \ldots, x_S, x_0, x_1, \ldots, x_S, \ldots$$

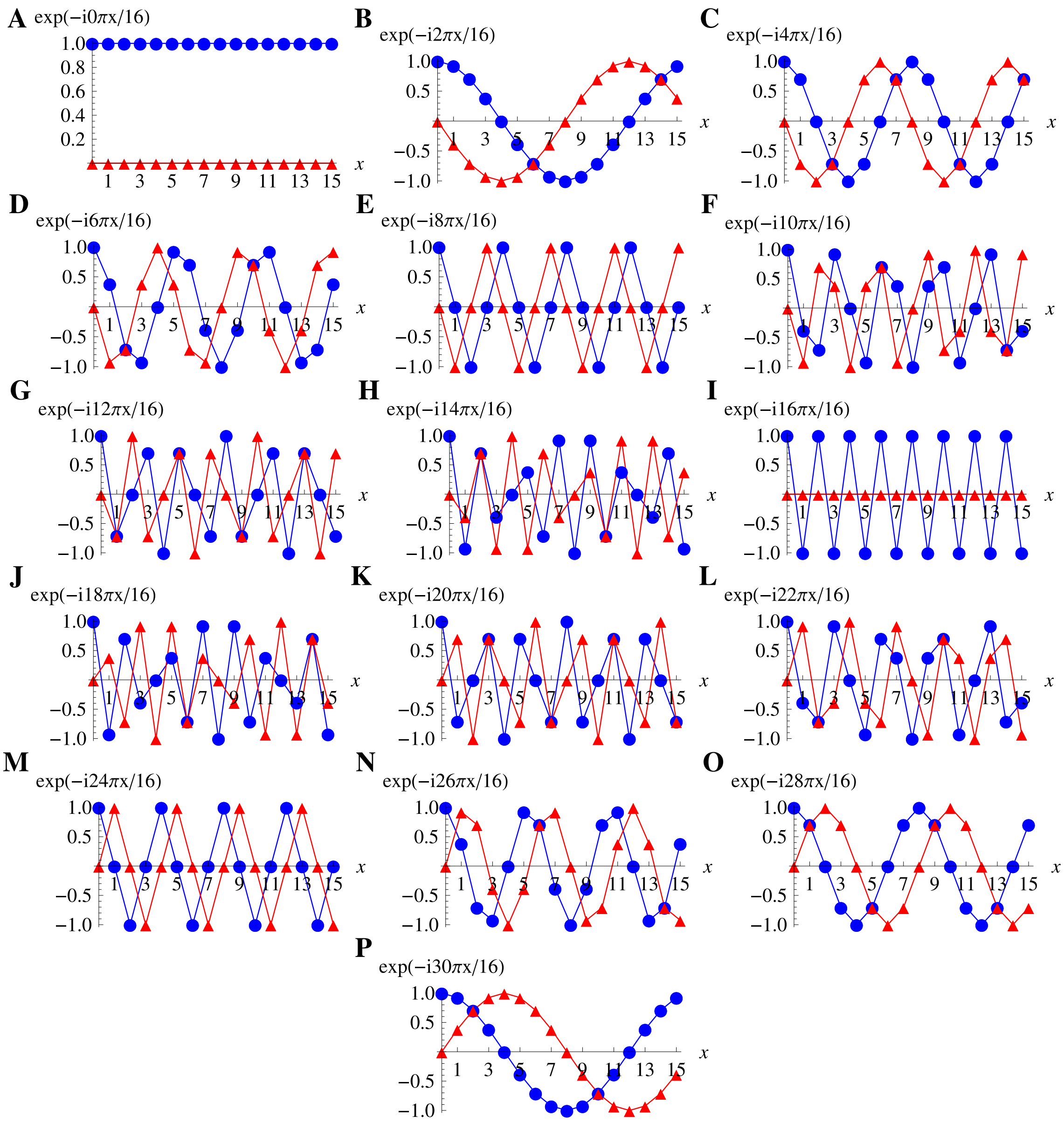

Fig. S3. Basis states for a 16-point discrete FT. Plots (**A**)-(**P**) depict 16 basis states $\exp\{-i2\pi k/(S+1)\}$ for $S = 15$ and $k = 0, \ldots, S$. Blue circles denote real, while red triangles – imaginary components of the states.

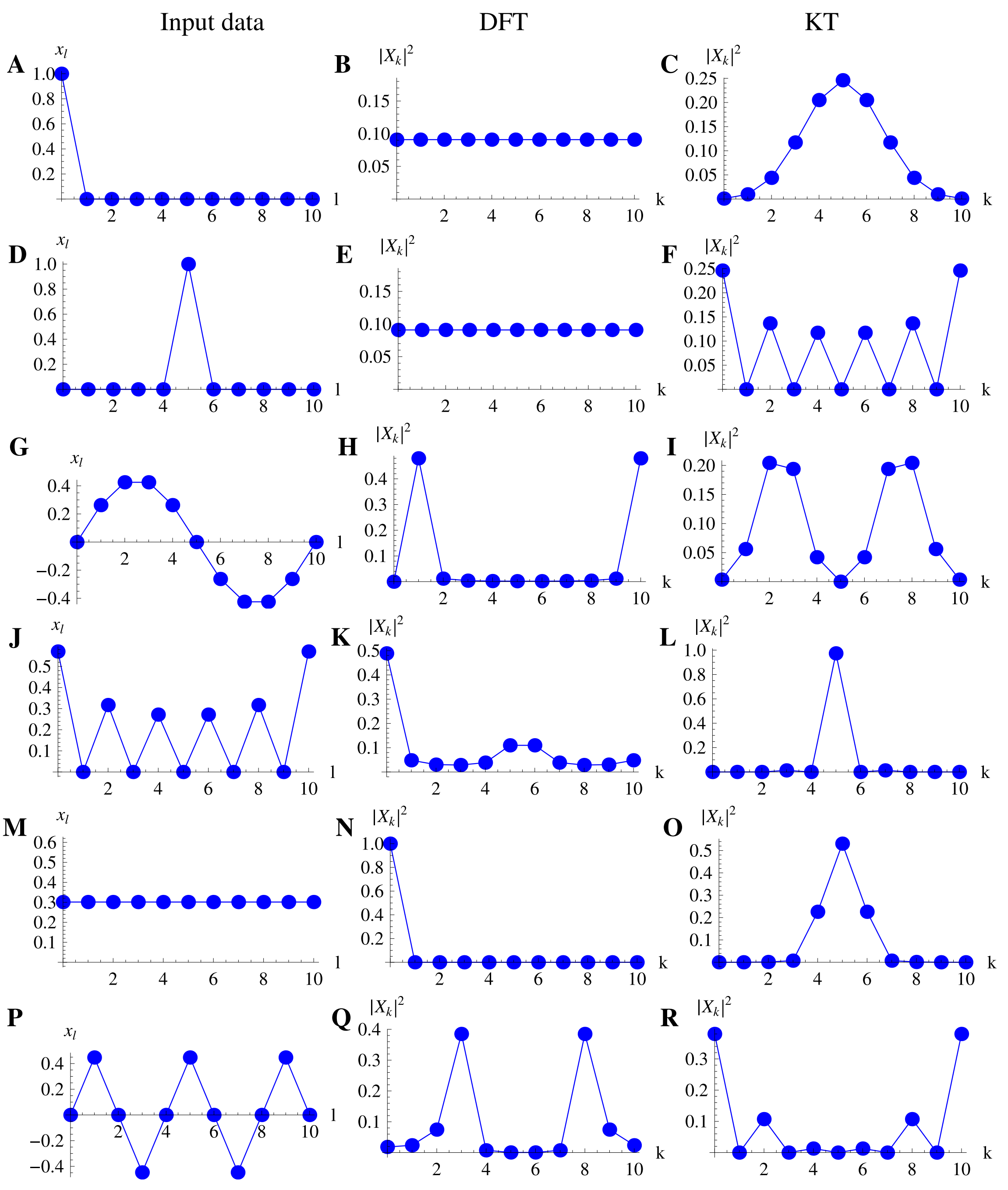

Fig. S4. KT versus DFT. The left column depicts exemplary input data (x_l) for $l = 0, \ldots,$ 10, while the middle and right columns – the results of computation of $|X_k|^2$ using the DFT and the Kravchuk transform, respectively.

and so is the resulting sequence

$$\ldots, X_0, X_1, \ldots, X_S, X_0, X_1, \ldots, X_S, X_0, X_1, \ldots, X_S, \ldots$$

As a result of this, two shifted input sequences, e.g. $(1,0,0,0,0,0,0,0,0)$ (Fig. S4(**A**)) and $(0,0,0,0,1,0,0,0,0)$ (Fig. S4(**D**)), are transformed by the DFT to the output differing only by a complex phase (Fig. S4(**B**) & (**E**)). Figs. S4 shows DFT and KT for exemplary input sequences.

α-fractional DFT (also known as DFRFT) for $0 < \alpha \leq 1$ is defined as (*9*)

$$X_k = \sqrt{\frac{\sin\theta - i\cos\theta}{S+1}} e^{\frac{i}{2}k^2\cot\theta} \sum_{l=0}^{S} e^{-i2\pi\frac{kl}{S+1}} e^{\frac{i}{2}l^2\cot\theta} \cdot x_l \tag{S18}$$

where $\theta = \frac{\pi\alpha}{2}$. For $\alpha = 1$ Eq. S18 reduces to Eq. S17.

1.5 KT and DFT software implementations

Algorithms for computation of Kravchuk polynomials and transforms as well as the FFT have been proposed both for software and hardware solutions. The most known library for computation of the KT is POLPAK, while for the FFT it is FFTW. KT algorithms underperform the FFT in speed because their number of operations is $O(n^2\log^2 n)$ compared to $O(n\log n)$ for the FFT. This result has been improved by Venkataramana et al. to $O(n^2)$ by using the Clenshaw's recurrence formula (*17*). Nevertheless, this seriously limits application of the KT in data processing. For example, an image of 512×512 pixels is transformed with the KT in 15 min instead of several seconds as is in case of the use of FFT (*31*).

2 Applications of Kravchuk transform

Following the seminal paper by Yap et al. (*15*), numerous applications of the Kravchuk functions, polynomials and the KT have been proposed.

2.1 Image processing

In computer image processing, image moments are vectors or matrices which describe interesting properties of the source data. They can be obtained e.g. by decomposition of pixel intensities in the basis of orthogonal functions. Kravchuk polynomials have been found to be extremely

useful for this purpose, as they are well defined on a finite domain of raster images and the computed moments carry out a lot of information about characteristic image features. In practice, computing Kravchuk moments is equivalent to performing the Kravchuk transform of the input image. This method has been already shown to be useful in optical character recognition (*15*), autonomous reading of the sign language, hand signature discrimination, writer identification, automatic face and gesture recognition (*32*) as well as facial expression and gait analysis. Importantly, this approach allows one to extract both local and global features of the input data and to recognize images corrupted with noise or tilt and possessing change in e.g. facial expression (*32*).

The α-fractional KT has been shown to be very useful in data watermarking schemes (watermark insertion and detection) (*33*). It allows to produce results which are invariant to image rotation, scaling and translation. For these reasons, the KT can be used in e.g. detection of copy-move forgery of images.

2.2 Search engines

The vectors obtained by the decomposition of the data in the basis of the Kravchuk functions have been also successfully tested in search engines. Such descriptors are especially able to capture sharp changes in the source data. By using only low-order Kravchuk moments one is able to perform e.g. search for similarly-looking 2- and 3-dimensional shapes (*34*), detect types of objects seen in a radar signal, perform automatic classification of videos, still images (including medical images) and sound files. Kravchuk transform has been also proposed as a method of efficient lossy compression of images, similarly to the discrete cosine transform used e.g. in JPEG file format.

2.3 Medical image recognition

In biology, Kravchuk image moments have been tested for determination of drugs in human plasma microscopic images and prediction of phosphorylation sites in cells. This approach has been thoroughly compared with the state-of-the-art methods towards various medical imaging applications (*35*). It was chosen as the best performing for breast mammography images, where it allowed to identify benign and malign masses with 90% accuracy, compared to 81% offered by the other techniques. This scheme was proven to outperform also other algorithms in analysis of computer tomography and ultrasound scans towards recognition of liver and prostate tumors.

2.4 Medical image reconstruction

KT has been already tested as a potential replacement of the FFT in generation of diagnostic ultrasound and magnetic resonance images (MRI). In these systems, the data regarding patients' bodies are collected in the frequency domain (k-space) and next, are processed by the inverse Fourier transform to obtain a real-space image. Tests were performed with MRI data coming from open repositories of brain and knee examinations. The images were reconstructed with the Kravchuk, Zernike, Pseudo-Zernike, Fourier-Merlin, Legendre and Chebyshev kernels (*15*, *35*). It has been pointed out that only the Kravchuk and Chebyshev transforms are discrete and allow to operate in the original Cartesian image coordinates. The Kravchuk-based method presented the best behavior in most test cases, giving the smallest reconstruction error and the highest peak signal-to-noise ratio as the moment order increased thus, is best suited for processing of high resolution data.

To assess the advantage of the KT for the MRI diagnostics, we performed similar steps to the ones presented in (*35*). Fig. S5 shows a comparative numerical study of the KT and FFT for a "pirate" test image and a brain scan from the OASIS database. Both source figures have resolution of 512×512 pixels. The source image of the brain is made of k-space raw data from an MRI system, while the "pirate" was originally prepared in the real space and next converted to the k-space using the NumPy numerical library to keep both data sets in the same form. A third data set consisted of the original brain scan but truncated to 256×256 values in the k-space by removing (zeroing) the higher-frequency components.

Subsequently, a 1% white Gaussian noise was added in parallel to all three data sets (still in the k-space). No further distortion was applied to any of the figures. Finally, the k-space images were transformed to the real space (reconstructed) with the FFT and KT.

In our test, the FFT produced artifacts, while some details (which could be tumor cells) were missing. This is best captured by the structural similarity index (SSIM). For the "pirate" it was 0.73 (FFT) and 0.92 (KT), and for the 512×512 brain it was 0.84 and 0.98, respectively. In case of the 256×256 brain figure, SSIM achieved was 0.72 for the FFT and 0.91 for the KT. The mean square error (MSE) was ten times smaller and the peak signal-to-noise ratio (PSNR) was 10 dB larger for the KT than for the FFT. The FFT led to degradation of the usable resolution from 1-2 mm per voxel to over 5 mm. Our findings confirmed the results of the previous research (*35*).

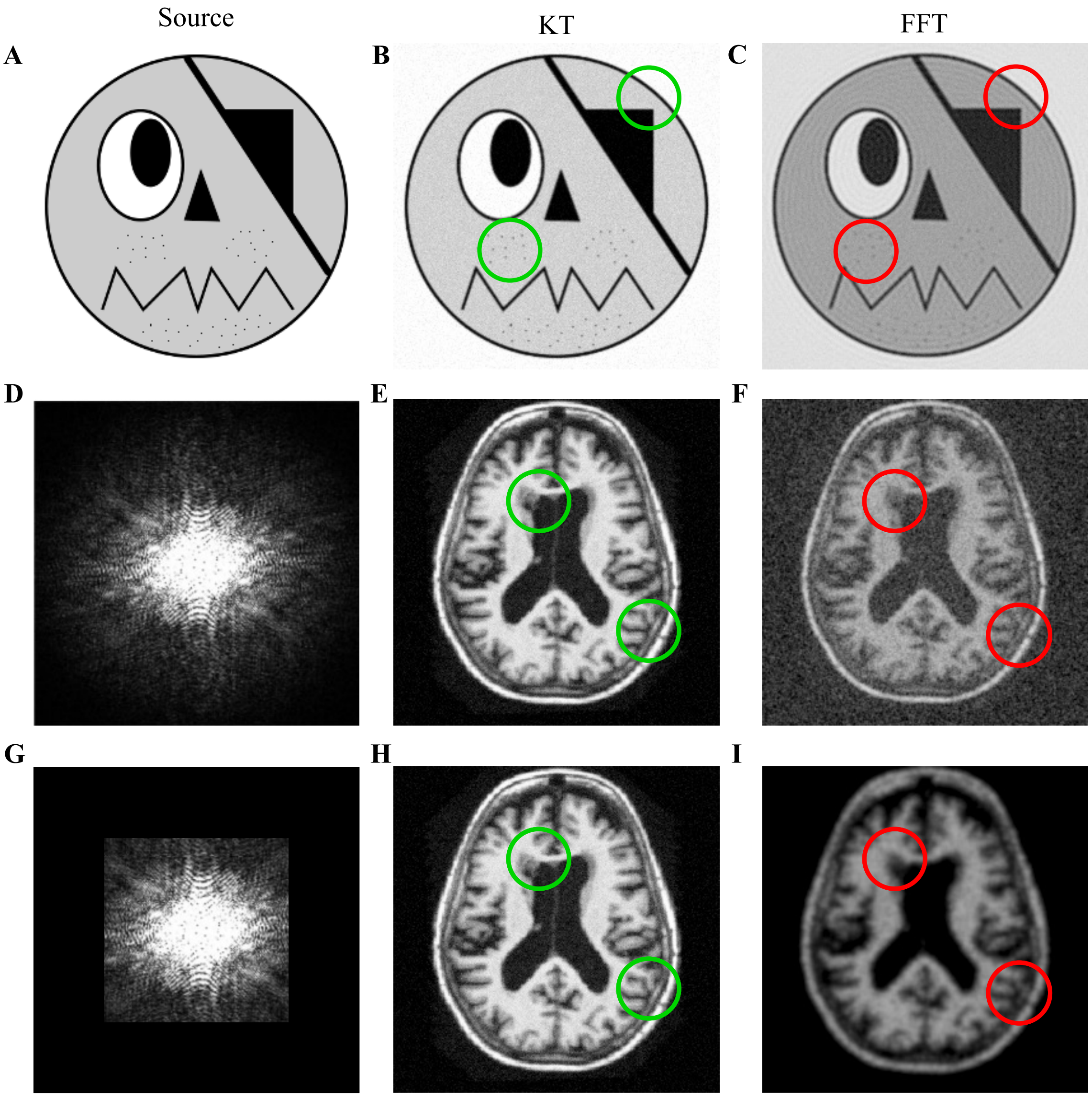

Fig. S5. Example of FFT and KT image processing. Two 512×512-pixel test images, a "pirate" (**A**) and a brain scan, the latter in a form of a raw k-space data from the OASIS database (**D**), were used. First, the "pirate" image has been transformed into the k-space with the FFT algorithm to keep both inputs in the same form. Additionally, a third set of data was created by truncating the brain raw data to 256×256 values by removing higher-frequency components (**G**). Next, all the images in the k-space were supplemented with a 1% additive white Gaussian noise, and reconstructed with corresponding inverse transforms to model the operation of an MRI analysis. (**B**), (**E**) & (**H**) are the images reconstructed with the KT. The green circles mark some fine details which were retained during this processing. (**C**), (**F**) & (**I**) are the images reconstructed using the FFT. The red circles highlight the artifacts.

3 Theory: multi-photon Hong–Ou–Mandel interference

We will now analyze in a detailed manner a generalized multi-photon HOM effect. As explained in the main text, we consider two interfering modes a and b on a beam splitter device with a tunable reflectivity r (defined as the probability of reflection of a single photon). As the input states we take photon number (Fock) states $|l\rangle_a = \frac{(a^\dagger)^l}{\sqrt{l!}} |0\rangle_a$ and $|S-l\rangle_b = \frac{(b^\dagger)^{S-l}}{\sqrt{(S-l)!}} |0\rangle_b$.

3.1 The Schwinger representation

One may represent the su(2) Lie algebra in terms of the annihilation and creation operators of the harmonic oscillator – the Schwinger representation. For a single spin two independent oscillators a and b are required. The spin operators are then constructed in the following way

$$S_x = \frac{a^\dagger b + a\, b^\dagger}{2}, \qquad S_y = \frac{i\left(a\, b^\dagger - a^\dagger b\right)}{2}, \qquad S_z = \frac{a^\dagger a - b^\dagger b}{2}, \qquad S_0 = \frac{a^\dagger a + b^\dagger b}{2} \tag{S19}$$

S_0 is the Casimir operator $S_0(S_0+1) = S_x^2 + S_y^2 + S_z^2$. The spin components fulfill the standard su(2) commutation relations

$$[S_x, S_y] = iS_z, \qquad [S_y, S_z] = iS_x, \qquad [S_z, S_x] = iS_y \tag{S20}$$

3.2 Beam splitter

Interference of two independent modes a and b on a beam splitter is governed by the following Hamiltonian

$$H = H_0 + H_{BS}, \tag{S21}$$

$$H_0 = \frac{\hbar}{2}\left(a^\dagger a + b^\dagger b\right) \tag{S22}$$

$$H_{BS} = \frac{i\hbar}{2}\left(a^\dagger b\, e^{-i\varphi} - a\, b^\dagger e^{i\varphi}\right) \tag{S23}$$

H_0 is the free quantum oscillator energy and H_{BS} – the beam splitter interaction (*19*). φ is the phase difference between the reflected and transmitted fields behind the beam splitter. H_0 commutes with H_{BS}.

Using the Schwinger representation, we express H in terms of the spin operators S_0, S_x, S_y, S_z

$$H_0 = \hbar S_0 \tag{S24}$$

$$a^\dagger b\, e^{-i\varphi} - a\, b^\dagger e^{i\varphi} = \cos\varphi\left(a^\dagger b - a\, b^\dagger\right) - i\sin\varphi\left(a^\dagger b + a\, b^\dagger\right) \tag{S25}$$

$$= 2i\left(\cos\varphi\cdot S_y - \sin\varphi\cdot S_x\right) \tag{S26}$$

$$H_{BS} = \tfrac{i\hbar}{2}\, 2i\left(\cos\varphi\cdot S_y - \sin\varphi\cdot S_x\right) \tag{S27}$$

$$= \hbar\left(\sin\varphi\cdot S_x - \cos\varphi\cdot S_y\right) \tag{S28}$$

The Hamiltonian generates the evolution operator

$$U = \exp\{-i\theta H/\hbar\} \tag{S29}$$

$$= \exp\{-i\theta(H_0 + H_{BS})/\hbar\} \tag{S30}$$

$$= \exp\{-i\theta\, H_{BS}/\hbar\}\exp\{-i\theta\, H_0/\hbar\} \tag{S31}$$

$$= U_{BS}\, U_0, \tag{S32}$$

$$U_0 = \exp\left\{-i\theta S_0\right\}, \tag{S33}$$

$$U_{BS} = \exp\left\{-i\theta\left(\sin\varphi\cdot S_x - \cos\varphi\cdot S_y\right)\right\} \tag{S34}$$

The evolution in the Heisenberg picture allows to establish a linear relation between the input (a, b) and the output (a_r, a_t) annihilation operators

$$a_r = U_{BS}^\dagger\, a\, U_{BS} = a\cos\tfrac{\theta}{2} + b\, e^{-i\varphi}\sin\tfrac{\theta}{2} \tag{S35}$$

$$a_t = U_{BS}^\dagger\, b\, U_{BS} = -a\, e^{i\varphi}\sin\tfrac{\theta}{2} + b\cos\tfrac{\theta}{2} \tag{S36}$$

The relation takes the following matrix form

$$\mathbf{U_{BS}} = \begin{pmatrix} \cos\frac{\theta}{2} & \sin\frac{\theta}{2}\, e^{-i\varphi} \\ -\sin\frac{\theta}{2} e^{i\varphi} & \cos\frac{\theta}{2} \end{pmatrix} \tag{S37}$$

where $\mathbf{U_{BS}}\,\mathbf{U_{BS}^\dagger} = \mathbf{1}$ and $\mathbf{U_{BS}^\dagger} = \mathbf{U_{BS}}^{-1}$ hold true. U_0 amounts to a global phase.

We now substitute $\sin\frac{\theta}{2} = \sqrt{r}$ and $\cos\frac{\theta}{2} = \sqrt{1-r}$ to relate the evolution directly to the beam splitter reflectivity

$$\mathbf{U_{BS}} = \begin{pmatrix} \sqrt{1-r} & e^{-i\varphi}\sqrt{r} \\ -e^{i\varphi}\sqrt{r} & \sqrt{1-r} \end{pmatrix} \tag{S38}$$

$$\mathbf{U_{BS}}^{-1} = \mathbf{U_{BS}^\dagger} = \begin{pmatrix} \sqrt{1-r} & -e^{-i\varphi}\sqrt{r} \\ e^{i\varphi}\sqrt{r} & \sqrt{1-r} \end{pmatrix} \tag{S39}$$

This brings us to the following relation between the input and output creation operators, to be used in the next section

$$\begin{pmatrix} a \\ b \end{pmatrix} = \mathbf{U_{BS}}^{-1} \begin{pmatrix} a_r \\ a_t \end{pmatrix} = \begin{pmatrix} \sqrt{1-r} & -e^{-i\varphi}\sqrt{r} \\ e^{i\varphi}\sqrt{r} & \sqrt{1-r} \end{pmatrix} \begin{pmatrix} a_r \\ a_t \end{pmatrix} \tag{S40}$$

$$\begin{pmatrix} a^\dagger \\ b^\dagger \end{pmatrix} = \begin{pmatrix} \sqrt{1-r} & -e^{i\varphi}\sqrt{r} \\ e^{-i\varphi}\sqrt{r} & \sqrt{1-r} \end{pmatrix} \begin{pmatrix} a_r^\dagger \\ a_t^\dagger \end{pmatrix} \tag{S41}$$

$$a^\dagger = \sqrt{1-r}\, a_r^\dagger - e^{i\varphi}\sqrt{r}\, a_t^\dagger, \tag{S42}$$

$$b^\dagger = e^{-i\varphi}\sqrt{r}\, a_r^\dagger + \sqrt{1-r}\, a_t^\dagger \tag{S43}$$

3.3 Photon number amplitude

Let the input states in modes a and b be the Fock states $|l\rangle$ and $|S-l\rangle$, respectively. Then,

$$U_0 \, |l\rangle_a \, |S-l\rangle_b = e^{-i\theta\frac{S}{2}} \, |l\rangle_a \, |S-l\rangle_b \,, \tag{S44}$$

$$U_{BS} \, |l\rangle_a \, |S-l\rangle_b = U_{BS} \frac{\left(a^\dagger\right)^l}{\sqrt{l!}} \frac{\left(b^\dagger\right)^{S-l}}{\sqrt{(S-l)!}} \, |0\rangle \tag{S45}$$

$$= \frac{1}{\sqrt{l!\,(S-l)!}} \left(\sqrt{1-r}\, a_r^\dagger - e^{i\varphi}\sqrt{r}\, a_t^\dagger\right)^l \left(e^{-i\varphi}\sqrt{r}\, a_r^\dagger + \sqrt{1-r}\, a_t^\dagger\right)^{S-l} |0\rangle \tag{S46}$$

$$= \frac{1}{\sqrt{l!\,(S-l)!}} \sum_{m=0}^{l} \sum_{n=0}^{S-l} \binom{l}{m} \binom{S-l}{n} \left(\sqrt{1-r}\, a_r^\dagger\right)^m \left(-e^{i\varphi}\sqrt{r}\, a_t^\dagger\right)^{l-m} \times$$
$$\times \left(e^{-i\varphi}\sqrt{r}\, a_r^\dagger\right)^n \left(\sqrt{1-r}\, a_t^\dagger\right)^{S-l-n} |0\rangle \tag{S47}$$

$$= \frac{1}{\sqrt{l!\,(S-l)!}} \sum_{m=0}^{l} \sum_{n=0}^{S-l} \binom{l}{m} \binom{S-l}{n} \left(-e^{i\varphi}\sqrt{r}\right)^l \left(\sqrt{1-r}\right)^{S-l} \times \tag{S48}$$
$$\times \left(\sqrt{1-r}\right)^{m-n} \left(\sqrt{r}\right)^{n-m} \left(e^{-i\varphi}\right)^{m+n} \times$$
$$\times (-1)^{-m} \left(a_r^\dagger\right)^{m+n} \left(a_t^\dagger\right)^{S-m-n} |0\rangle$$

$$U\left|l\right\rangle_a\left|S-l\right\rangle_b = e^{-i\theta\frac{S}{2}}\frac{\left(-e^{i\varphi}\sqrt{r}\right)^l\left(\sqrt{1-r}\right)^{S-l}}{\sqrt{l!\,(S-l)!}}\sum_{m=0}^{l}\sum_{n=0}^{S-l}\binom{l}{m}\binom{S-l}{n}(-1)^{-m}\times$$
$$\times\left(e^{-i\varphi}\right)^{m+n}\left(\sqrt{\tfrac{1-r}{r}}\right)^{m-n}\times$$
$$\times\sqrt{(m+n)!\,(S-m-n)!}\left|m+n,S-m-n\right\rangle \tag{S49}$$

Let us substitute $m+n=k$ to change the summation variables. Then

$$\left|m+n,S-m-n\right\rangle = \left|k,S-k\right\rangle$$

and the ranges of k and m are as follows

$$0\leq m+n=k\leq S$$
$$0\leq k-m=n\leq S-l\Rightarrow k+l-S\leq m\leq k$$

$$\sum_{m=0}^{l}\sum_{n=0}^{S-l}\Rightarrow\sum_{k=0}^{S}\sum_{m=\max\{0,k+l-S\}}^{\min\{l,k\}}$$

$$U\left|l\right\rangle_a\left|S-l\right\rangle_b = e^{-i\theta\frac{S}{2}}\frac{\left(-e^{i\varphi}\sqrt{r}\right)^l\left(\sqrt{1-r}\right)^{S-l}}{\sqrt{l!\,(S-l)!}}\sum_{k=0}^{S}\sum_{m=\max\{0,k+l-S\}}^{\min\{l,k\}}\binom{l}{m}\binom{S-l}{k-m}(-1)^{-m}\times$$
$$\times\left(e^{-i\varphi}\right)^{k}\left(\sqrt{\tfrac{1-r}{r}}\right)^{2m-k}\sqrt{k!\,(S-k)!}\left|k,S-k\right\rangle \tag{S50}$$

The probability amplitude of detecting k and $S-k$ photons behind the beam splitter provided that l and $S-l$ were injected into it is

$$\mathcal{A}_S(k,l) = \left\langle k,S-k\right|U\left|l,S-l\right\rangle \tag{S51}$$

thus

$$U\left|l\right\rangle_a\left|S-l\right\rangle_b = \sum_{k=0}^{S}\mathcal{A}_S(k,l)\left|k,S-k\right\rangle \tag{S52}$$

where

$$\mathcal{A}_S(k,l) = e^{-i\theta\frac{S}{2}} \frac{\left(-e^{i\varphi}\sqrt{r}\right)^l \left(\sqrt{1-r}\right)^{S-l}}{\sqrt{l!\,(S-l)!}} \sum_{m=\max\{0,k+l-S\}}^{\min\{l,k\}} \binom{l}{m}\binom{S-l}{k-m} (-1)^{-m} \left(e^{-i\varphi}\right)^k \times$$
$$\times \left(\sqrt{\tfrac{1-r}{r}}\right)^{2m-k} \sqrt{k!\,(S-k)!} \tag{S53}$$

$$= e^{-i\theta\frac{S}{2}} \frac{\left(-e^{i\varphi}\sqrt{r}\right)^l \left(\sqrt{1-r}\right)^{S-l}}{\sqrt{l!\,(S-l)!}} \left(e^{-i\varphi}\right)^k \left(\sqrt{\tfrac{1-r}{r}}\right)^{-k} \sqrt{k!\,(S-k)!} \times$$
$$\times \sum_{m=\max\{0,k+l-S\}}^{\min\{l,k\}} \binom{l}{m}\binom{S-l}{k-m} (-1)^{-m} \left(\tfrac{1-r}{r}\right)^m \tag{S54}$$

The inner sum over m in Eq. S54 is a hypergeometric series. In order to simplify it, the identities from Section 4 are used. The four cases below (A-D) correspond to different summation ranges. For simplicity, let us assume that $l \leq S-l$, i.e. $l \leq \frac{S}{2}$.

Case A: $\min\{l,k\} = l$ and $\max\{0, k+l-S\} = 0$. This implies $l \leq k \leq S-l$

$$\sum_{m=0}^{l} \binom{l}{m}\binom{S-l}{k-m} (-1)^{-m} \left(\tfrac{1-r}{r}\right)^m \tag{S55}$$

$$= \sum_{m=0}^{l} \binom{l}{m}\binom{S-l}{k-m} \left(1-\tfrac{1}{r}\right)^m \tag{S56}$$

$$= \sum_{m=0}^{l} \binom{l}{m} \frac{(S-l)!}{(k-m)!\,(S-l-k+m)!} \frac{(S-l-k)!\,k!}{(S-l-k)!\,k!} \left(1-\tfrac{1}{r}\right)^m \tag{S57}$$

$$= \sum_{m=0}^{l} \binom{l}{m} \frac{(S-l)!}{(S-l-k)!\,k!} \frac{(S-l-k)!}{(S-l-k+m)!} \frac{k!}{(k-m)!} \left(1-\tfrac{1}{r}\right)^m \tag{S58}$$

$$= \sum_{m=0}^{l} \binom{l}{m}\binom{S-l}{k} \frac{(-1)^m(-k)_k}{(S-l-k+1)_k} \left(1-\tfrac{1}{r}\right)^m \qquad \text{cf. (S110), (S111)} \tag{S59}$$

$$= \binom{S-l}{k} {}_2F_1\left[-l,-k;S-l-k+1;1-\tfrac{1}{r}\right] \qquad \text{cf. (S113)} \tag{S60}$$

$$= \binom{S-l}{k} \frac{(S-l-k+1+k)_K}{(S-l-k+1)_K} {}_2F_1\left[-l,-k;-S;\tfrac{1}{r}\right] \quad \text{cf. (S114)} \tag{S61}$$

$$= \binom{S}{k} {}_2F_1\left[-l,-k;-S;\tfrac{1}{r}\right] \tag{S62}$$

Case B: $\min\{l,k\} = k$ and $\max\{0, k+l-S\} = k+l-S$. This implies $S-l \le k \le l$, i.e. the empty set.

Case C: $\min\{l,k\} = k$ and $\max\{0, k+l-S\} = 0$. This implies $k \le l \le S-l$

$$\sum_{m=0}^{k} \binom{l}{m}\binom{S-l}{k-m}(-1)^{-m}\left(\tfrac{1-r}{r}\right)^m \tag{S63}$$

$$= \sum_{m=0}^{k} \binom{l}{m}\binom{S-l}{k-m}\left(1-\tfrac{1}{r}\right)^m \tag{S64}$$

$$= \sum_{m=0}^{k} \frac{l!}{m!\,(l-m)!}\frac{(S-l)!}{(k-m)!\,(S-l-k+m)!}\frac{k!\,(S-l-k)!}{k!\,(S-l-k)!}\left(1-\tfrac{1}{r}\right)^m \tag{S65}$$

$$= \sum_{m=0}^{k} \frac{k!}{m!\,(k-m)!}\frac{(S-l)!}{k!\,(S-l-k)!}\frac{l!}{(l-m)!}\frac{(S-l-k)!}{(S-l-k+m)!}\left(1-\tfrac{1}{r}\right)^m \tag{S66}$$

$$= \sum_{m=0}^{k} \binom{k}{m}\binom{S-l}{k}\frac{(-1)^m(-l)_k}{(S-l-k+1)_k}\left(1-\tfrac{1}{r}\right)^m \quad \text{cf. (S110), (S111)} \tag{S67}$$

$$= \binom{S-l}{k} {}_2F_1\left[-k,-l;S-l-k+1;1-\tfrac{1}{r}\right] \quad \text{cf. (S113)} \tag{S68}$$

$$= \binom{S-l}{k} {}_2F_1\left[-l,-k;S-l-k+1;1-\tfrac{1}{r}\right] \quad \text{cf. (S112)} \tag{S69}$$

$$= \binom{S-l}{k} \frac{(S-l-k+1+k)_K}{(S-l-k+1)_K} {}_2F_1\left[-l,-k;-S;\tfrac{1}{r}\right] \quad \text{cf. (S114)} \tag{S70}$$

$$= \binom{S}{k} {}_2F_1\left[-l,-k;-S;\tfrac{1}{r}\right] \tag{S71}$$

Case D: $\min\{l,k\}=l$ and $\max\{0,k+l-S\}=k+l-S$. This implies $l\leq S-l\leq k$. To compute the sum, the following substitution is used: $m=l-m'$

$$\sum_{m=k+l-S}^{l}\binom{l}{m}\binom{S-l}{k-m}\left(1-\tfrac{1}{r}\right)^{m}=\sum_{m'=0}^{S-k}\binom{l}{l-m'}\binom{S-l}{k+m'-l}\left(1-\tfrac{1}{r}\right)^{l-m'} \tag{S72}$$

$$=\left(1-\tfrac{1}{r}\right)^{l}\sum_{m'=0}^{S-k}\binom{S-k}{m'}\frac{m'!\,(S-k-m')!}{(S-k)!}\frac{l!}{(l-m')!\,m'!}\times \tag{S73}$$

$$\times\frac{(S-l)!}{(S-k-m')!\,(k+m'-l)!}(-1)^{-m'}\left(\tfrac{1}{r}-1\right)^{-m'}$$

$$=\left(1-\tfrac{1}{r}\right)^{l}\sum_{m'=0}^{S-k}\binom{S-k}{m'}(-1)^{m'}\frac{l!}{(l-m')!}\frac{(S-l)!}{(S-k)!\,(k+m'-l)!}\left(\tfrac{1}{r}-1\right)^{-m'} \tag{S74}$$

$$=\left(1-\tfrac{1}{r}\right)^{l}\sum_{m'=0}^{S-k}\binom{S-k}{m'}(-1)^{m'}(-1)^{m'}(-l)_{m'}\times \tag{S75}$$

$$\times\frac{(S-l)!}{(S-k)!\,(k-l+m')!}\frac{(k-l)!}{(k-l)!}\left(\tfrac{r}{1-r}\right)^{m'}$$

$$=\left(1-\tfrac{1}{r}\right)^{l}\sum_{m'=0}^{S-k}\binom{S-k}{m'}(-1)^{m'}(-l)_{m'}\frac{(S-l)!}{(S-k)!\,(S-l-S+k)!}\times \quad \text{cf. (S110), (S111)}$$

$$\times\frac{(k-l)!}{(k-l+m')!}\left(\tfrac{r}{r-1}\right)^{m'} \tag{S76}$$

$$=\left(1-\tfrac{1}{r}\right)^{l}\binom{S-l}{S-k}\sum_{m'=0}^{S-k}\binom{S-k}{m'}(-1)^{m'}\frac{(-l)_{m'}}{(k-l+1)_{m'}}\left(\tfrac{r}{r-1}\right)^{m'} \quad \text{cf. (S113)} \tag{S77}$$

$$=\left(1-\tfrac{1}{r}\right)^{l}\binom{S-l}{S-k}{}_2F_1\left[-(S-k),-l;k-l+1;\tfrac{r}{r-1}\right] \quad \text{cf. (S114)} \tag{S78}$$

$$=\left(1-\tfrac{1}{r}\right)^{l}\binom{S-l}{S-k}\frac{(k-l+1+l)_{S-k}}{(k-l+1)_{S-k}}\times \tag{S79}$$

$$\times\,{}_2F_1\left[-(S-k),-l;1-S+k-l-(k-l+1);\tfrac{-1}{r-1}\right]$$

$$=\left(1-\tfrac{1}{r}\right)^{l}\binom{S}{S-k}{}_2F_1\left[-(S-k),-l;-S;\tfrac{-1}{r-1}\right] \quad \text{cf. (S115)} \tag{S80}$$

$$= \underbrace{\left(1-\tfrac{1}{r}\right)^l \left(1-\tfrac{-1}{r-1}\right)^l}_{=1} \binom{S}{k} {}_2F_1 \left[-S+(S-k), -l; -S; \frac{\frac{-1}{r-1}}{\frac{-1}{r-1}-1}\right] \tag{S81}$$

$$= \binom{S}{k} {}_2F_1 \left[-k, -l; -S; \tfrac{1}{r}\right] \quad \text{cf. (S112)} \tag{S82}$$

$$= \binom{S}{k} {}_2F_1 \left[-l, -k; -S; \tfrac{1}{r}\right] \tag{S83}$$

Summarizing, the inner sum in Eq. S54 equals $\binom{S}{k}{}_2F_1\left[-l,-k;-S;\frac{1}{r}\right]$ under the assumption that $l \leq \frac{S}{2}$. The probability amplitude can be rewritten into the following form

$$\mathcal{A}_S(k,l) = \frac{\left(-e^{i\varphi}\sqrt{r}\right)^l \left(\sqrt{1-r}\right)^{S-l}}{\sqrt{l!\,(S-l)!}} e^{-i\theta\frac{S}{2}} \left(e^{-i\varphi}\right)^k \left(\sqrt{\tfrac{1-r}{r}}\right)^{-k} \sqrt{k!\,(S-k)!} \binom{S}{k} {}_2F_1 \left[-l, -k; -S; \tfrac{1}{r}\right] \tag{S84}$$

$$\sqrt{\frac{k!\,(S-k)!}{l!\,(S-l)!}} \binom{S}{k} = \sqrt{\binom{S}{k}\binom{S}{l}}$$

$$\mathcal{A}_S(k,l) = \sqrt{\binom{S}{k}\binom{S}{l}} (-1)^l \left(e^{i\varphi}\right)^{l-k} e^{-i\theta\frac{S}{2}} \left(\sqrt{1-r}\right)^S \left(\sqrt{\tfrac{r}{1-r}}\right)^{l+k} {}_2F_1 \left[-l, -k; -S; \tfrac{1}{r}\right] \tag{S85}$$

$$= \sqrt{\binom{S}{k}\binom{S}{l}} (-1)^l \left(e^{i\varphi}\right)^{l-k} e^{-i\theta\frac{S}{2}} \left(\cos\tfrac{\theta}{2}\right)^S \left(\tan\tfrac{\theta}{2}\right)^{l+k} {}_2F_1 \left[-l, -k; -S; \left(\sin\tfrac{\theta}{2}\right)^{-2}\right] \tag{S86}$$

$$= \sqrt{\binom{S}{k}\binom{S}{l}} (-1)^l \left(e^{i\varphi}\right)^{l-k} e^{-i\theta\frac{S}{2}} \left(\cos\tfrac{\theta}{2}\right)^S \left(\tan\tfrac{\theta}{2}\right)^{l+k} {}_2F_1 \left[-k, -l; -S; \left(\sin\tfrac{\theta}{2}\right)^{-2}\right] \tag{S87}$$

The photon number statistics behind the beam splitter is given by the probability $p_S(k,l) = |\mathcal{A}_S(k,l)|^2$

$$p_S(k,l) = \binom{S}{k}\binom{S}{l} \left(\cos\tfrac{\theta}{2}\right)^{2S} \left(\tan\tfrac{\theta}{2}\right)^{2(l+k)} \left|{}_2F_1 \left[-l, -k; -S; \left(\sin\tfrac{\theta}{2}\right)^{-2}\right]\right|^2 = p_S(l,k) \tag{S88}$$

3.4 Kravchuk transform

The α-fractional Kravchuk transform of an input sequence $x_n = f(\xi_n)$, where $n = 0, 1, \ldots, N$ and $\xi_n = (n - N/2)$, is defined as follows (*4*) (cf. Eq. 5.2)

$$\mathbf{X}_n = \sum_{n'=0}^{N} F^{\alpha}_{n,n'}\, x_{n'} \tag{S89}$$

$$F^{\alpha}_{n,n'} = e^{i\frac{\pi}{2}(n+n'-N\alpha/2)} \sqrt{\binom{N}{n}\binom{N}{n'}} \cos^N\left(\tfrac{\pi\alpha}{4}\right) \tan^{n+n'}\left(\tfrac{\pi\alpha}{4}\right) {}_2F_1\left[-n, -n'; -N; \sin^{-2}\left(\tfrac{\pi\alpha}{4}\right)\right] = F^{\alpha}_{n',n} \tag{S90}$$

$$= e^{i\frac{\pi}{2}(n'-n-N\alpha/2)} \sqrt{\frac{n!\,(N-n)!}{n'!\,(N-n')!}} \sin^{n'-n}\left(\tfrac{\pi\alpha}{4}\right) \cos^{N-n'-n}\left(\tfrac{\pi\alpha}{4}\right)\, k_n^{[\sin^2(\pi\alpha/4)]}(n', N) \tag{S91}$$

$$= e^{i\frac{\pi}{2}(n'-n-N\alpha/2)} \phi_n^{(p)}(n' - Np, N) \tag{S92}$$

where $k_n^{(p)}(n', N)$ is a Kravchuk polynomial and $\phi_n^{(p)}(n' - Np, N)$ is a Kravchuk function.

We used the following relations (*36*)

$$k_n^{(p)}(n', N) = (-1)^n \binom{N}{n} p^n\, {}_2F_1\left[-n, -n'; -N; \tfrac{1}{p}\right] \tag{S93}$$

$$\phi_n^{(p)}(n' - Np, N) = \sqrt{\frac{n!\,(N-n)!}{n'!\,(N-n')!}} \sqrt{p^{n'-n}(1-p)^{N-n-n'}}\, k_n^{(p)}(n', N) \tag{S94}$$

$$\phi_n^{(p)}(n' - Np, N) = (-1)^{n+n'} \phi_{n'}^{(p)}(n - Np, N) \tag{S95}$$

as well as the fact that the Kravchuk functions are orthonormal

$$\sum_{n'=0}^{N} \phi_n^{(p)}(n' - Np, N)\, \phi_m^{(p)}(n' - Np, N) = \delta_{n,m} \tag{S96}$$

Now we turn $\mathcal{A}_S(k, l)$ shown in Eq. S87 to the form of Eq. S92

$$\boxed{\begin{aligned} \mathcal{A}_S(k, l) &= e^{-i\theta\frac{S}{2}}\, e^{i\varphi(l-k)}\, (-1)^{k+l}\, \phi_k^{(r)}(l - Sr, S) \\ &= e^{i\frac{\pi}{2}\left(\frac{2(\pi+\varphi)}{\pi}(l-k) - S\frac{\theta}{\pi}\right)}\, \phi_k^{(r)}(l - Sr, S) \\ &= e^{-i\theta\frac{S}{2}}\, e^{i\varphi(l-k)}\, \phi_l^{(r)}(k - Sr, S) \\ &= e^{i\frac{\pi}{2}\left(\frac{2\varphi}{\pi}(l-k) - S\frac{\theta}{\pi}\right)}\, \phi_l^{(r)}(k - Sr, S), \end{aligned}} \tag{S97}$$

where $r = \sin^2 \frac{\theta}{2}$.

In specific, if we take $\varphi = -\frac{\pi}{2}$ and rearrange terms

$$\mathcal{A}_S(k,l) = e^{i\frac{\pi}{2}(k+l-S\frac{\theta}{\pi})}\sqrt{\binom{S}{k}\binom{S}{l}}\left(\cos\tfrac{\theta}{2}\right)^S\left(\tan\tfrac{\theta}{2}\right)^{l+k}{}_2F_1\left[-k,-l;-S;\left(\sin\tfrac{\theta}{2}\right)^{-2}\right] \tag{S98}$$

$$= F_{k,l}^{\frac{2\theta}{\pi}} \tag{S99}$$

$$= e^{i\frac{\pi}{2}(l-k-S\frac{\theta}{\pi})}\phi_k^{\left(\sin^2\frac{\theta}{2}\right)}(l - S\sin^2\tfrac{\theta}{2}, S) \tag{S100}$$

$$\boxed{\mathcal{A}_S(k,l) = e^{i\frac{\pi}{2}(l-k-S\frac{\theta}{\pi})}\phi_k^{(r)}(l-Sr,S).} \tag{S101}$$

3.5 Quantum Kravchuk transform on a beam splitter

Let us send a superposition $\sum_{l=0}^{S} x_l\,|l, S-l\rangle$ to a BS. The superposition amplitudes encode the sequence $(x_1, \ldots, x_S)$ to be transformed. We will compute the probabilities of detecting $|k\rangle$ and $|S-k\rangle$ photons behind the BS

$$\left|\langle k,S-k|U_0\,U_{BS}\left(\sum_{l=0}^{S}x_l\cdot|l,S-l\rangle\right)\right|^2 = \left|\sum_{l=0}^{S}x_l\cdot\langle k,S-k|U_0\,U_{BS}|l,S-l\rangle\right|^2 \tag{S102}$$

$$= \left|\sum_{l=0}^{S}x_l\cdot e^{-i\theta\frac{S}{2}}\langle k,S-k|U_{BS}|l,S-l\rangle\right|^2 \tag{S103}$$

$$= \left|\sum_{l=0}^{S}x_l\cdot\mathcal{A}_S^{(r)}(k,l)\right|^2 \tag{S104}$$

$$= \left|\sum_{l=0}^{S}x_l\cdot e^{-i\theta\frac{S}{2}}e^{i\frac{\pi}{2}(l-k)}\phi_k^{(r)}(l-Sr,S)\right|^2 \tag{S105}$$

$$= |\mathrm{X}_k|^2 \tag{S106}$$

It is clear now that multi-photon interference on a beam splitter followed by photon-counting detection implements $\alpha = \frac{2\theta}{\pi}$-fractional QKT of the input probability amplitudes

$$(x_0, x_1, \ldots, x_S) \rightarrow (|\mathrm{X}_0|^2, |\mathrm{X}_1|^2, \ldots, |\mathrm{X}_S|^2) \tag{S107}$$

where $|\mathrm{X}_k|^2$ are experimentally determined photon number statistics for $k = 0, \ldots, S$.

4 Gauss hypergeometric function

Definition. The Gauss hypergeometric function is a special function defined with the following hypergeometric series

$$ {}_2F_1(a,b;c;z) = \sum_{k=0}^{\infty} \frac{(a)_k\,(b)_k}{(c)_k}\,\frac{z^k}{k!} \tag{S108}$$

where a, b and c are parameters, z is an argument and $(x)_k$ is the Pochhammer symbol

$$(x)_k = x(x+1)(x+2)\cdots(x+k-1) \tag{S109}$$

In general, all ${}_2F_1$ arguments and the parameter may be complex, $a, b, c, z \in \mathbb{C}$ however, within this note the arguments are always integer, $a, b, c \in \mathbb{Z}$ and the parameter is real, $z \in \mathbb{R}$.

Properties. The Pochhammer symbol can be expressed as a division of factorials

$$\frac{a!}{(a-k)!} = (-1)^k(-a)_k \tag{S110}$$

$$\frac{a!}{(a+k)!} = \frac{1}{(a+1)_k} \tag{S111}$$

The form of Eq. S108 implies that the arguments a and b can be swapped

$$ {}_2F_1(a,b;c;z) = \sum_{k=0}^{\infty} \frac{(a)_k\,(b)_k}{(c)_k}\,\frac{z^k}{k!} = \sum_{k=0}^{\infty} \frac{(b)_k\,(a)_k}{(c)_k}\,\frac{z^k}{k!} = {}_2F_1(b,a;c;z) \tag{S112}$$

In case of a negative a or b, the infinite sum in Eq. S108 is truncated because $(x)_k = 0$ if x is a negative integer and $k > -x$. Let us assume that $a < 0$ and $b \geq 0 \vee b < a$. Then, let $m = -a$

$$\begin{aligned} {}_2F_1(-m,b;c;z) &= \sum_{k=0}^{\infty} \frac{(-m)_k\,(b)_k}{(c)_k}\,\frac{z^k}{k!} && \text{cf. (S110)} \\ &= \sum_{k=0}^{m} (-1)^k \frac{m!}{k!\,(m-k)!}\,\frac{(b)_k}{(c)_k}\,z^k \\ &= \sum_{k=0}^{m} \binom{m}{k} (-1)^k \frac{(b)_k}{(c)_k} z^k \end{aligned} \tag{S113}$$

Moreover, for the same assumptions as in case of Eq. S113, the following transformation can be used to change z to $1-z$ [NIST Digital Library of Mathematical Functions, 15.8.7]

$$ {}_2F_1(-m,b;c;z) = \frac{(c-b)_m}{(c)_m}\,{}_2F_1(-m,b;b-c-m+1;1-z) \tag{S114}$$

Identities analogous to Eqs. S113 and S114 are also valid for negative b and $a \geq 0 \vee a < b$, due to Eq. S112.

Finally, the following Pfaff's hypergeometric transformation is valid for any a, b, c and z

$$ {}_2F_1(a, b; c; z) = (1 - z)^{-b} {}_2F_1(c - a, b; c; z/(z - 1)) \tag{S115} $$

5 Characterization of the setup

In order to estimate transmission losses, we performed Klyshko efficiency measurements on the setup. In a Klyshko measurement with one SPDC source and binary detectors, one counts single events C_A, C_B from either output channel and coincidence clicks C_{AB} between both channels and defines the Klyshko efficiencies η_A and η_B

$$ \eta_B = \frac{C_{AB}}{C_A} \tag{S116} $$

and vice versa. For low pump powers, these Klyshko efficiencies show a linear pump power dependency, and their intercept is a measure at zero pump power of total transmission efficiency (including both propagation and detection losses) of the associated spatial mode (*37*).

We pumped each of our SPDC sources, one at a time, with the variable beam-splitter in position $50 : 50$, at successively lower power values. The resulting four-mode correlated photon statistics were then transformed into binary "photon(s)/no-photon" datasets to emulate standard binary detectors such as avalanche photo-diodes, and we determined the total efficiencies of the heralding modes to be $\eta_1 = 50.3\%$ and $\eta_4 = 48.5\%$. The beam-splitter modes, carrying each a 3 dB loss from the splitter itself and an additional 1 dB due to splitter insertion loss and fiber-to-fiber coupling loss, exhibit a total efficiency of $\eta_2 = 21.6\%$ and $\eta_3 = 20.6\%$. Taking into account the additional optical elements in the splitter modes, the efficiencies are consistent. We account for the transmission losses of approximately $50\% \approx 3$ dB with 1 dB initial fiber in-coupling loss due to spatial mode mismatch, 0.25 dB from imperfect detectors, and the rest from three FC/PC fiber-to-fiber couplers per mode as well as bending losses in the transmission fibers between the experimental setup and the detectors.

Fig. S6 shows the standard HOM interference dip between both sources measured with binary detectors (InGaAs APDs) for a small mean photon number of the order of 10^{-4} in order to test the setup. The maximal visibility achieved is $V_{\text{HOM}} = 85.9\%$. An independent measurement of the second order correlation function for each SPDC source $g^{(2)} = \frac{\langle n^2 \rangle - \langle n \rangle}{\langle n \rangle^2} \geq 1.86 \approx$

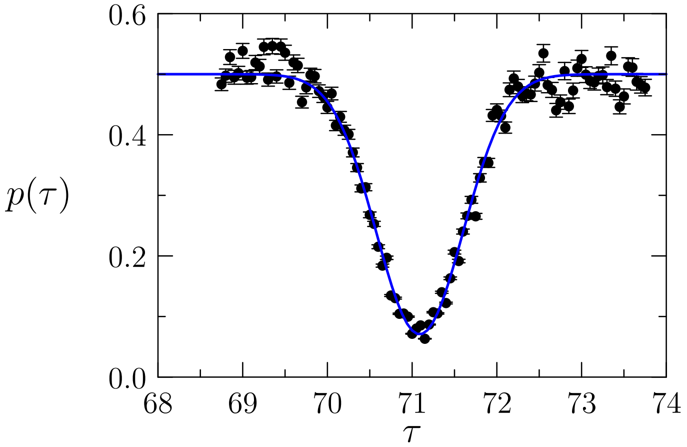

Fig. S6. HOM dip. Black dots with error bars represent the experimental results whereas the blue line is a fitted curve. The maximal visibility amounts to 85.9% which proves the quantum nature of impinging multiphoton states.

$1 + V_{\text{HOM}}$ is consistent with this result. From this, we can infer an effective Schmidt mode number of $K = \frac{1}{g^{(2)}-1} = 1.16$, (*38*) i.e. both of our SPDC sources are close to being single-mode.

The TES detectors used in the experiment were thoroughly characterized with quantum tomography methods (*20*). Their quantum efficiency is above 90%.

6 Analysis of the experimental data

6.1 HOM visibilities

The second-order visibility exceeding the classical value of 50% certifies quantum nature of the HOM interference and thus, the fractional QKT. The visibility is computed with the following formula (*39*)

$$v^{(2)} = \frac{n_{\text{max}} - n_{\text{min}}}{n_{\text{max}} + n_{\text{min}}} \tag{S117}$$

where n_{max} and n_{min} are the maximal and minimal number of events registered by the TES detectors for the given photon number S.

The obtained values are gathered in Tab. S1. For $S = 5$ it was always greater than 50%. The visibility of interference of $|1, 1\rangle$ given in this Table is much lower than the one reported in Fig. S6. This is because in order to perform quantum simulations with $S > 2$ we increased the power of a laser pumping our source. However, this power increase is too small to affect

Table S1. Second-order interferometric visibilities in HOM interference. The visibility above 50% proves quantum character of the interference. Two-mode Fock states $|\psi\rangle$ impinging on a beam splitter of a variable reflectivity r implement the fractional QKTs. $\bar{n}$ denotes the mean number of interfering photons reached in the experiment.

$\|\psi\rangle$	$r = 0.05$	$r = 0.3$	$r = 0.5$	$r = 0.95$
$\|0,1\rangle$	$87.2\% \pm 0.1\%$ ($\bar{n} = 0.2134$)	$35.1\% \pm 0.1\%$ ($\bar{n} = 0.2082$)	$0.8\% \pm 0.0\%$ ($\bar{n} = 0.2097$)	$87.8\% \pm 0.1\%$ ($\bar{n} = 0.2082$)
$\|0,2\rangle$	$98.2\% \pm 0.3\%$ ($\bar{n} = 0.2134$)	$59.9\% \pm 0.2\%$ ($\bar{n} = 0.2082$)	$26.5\% \pm 0.1\%$ ($\bar{n} = 0.2097$)	$99.0\% \pm 0.3\%$ ($\bar{n} = 0.2082$)
$\|0,3\rangle$	$99.7\% \pm 0.8\%$ ($\bar{n} = 0.2134$)	$78.6\% \pm 0.7\%$ ($\bar{n} = 0.2082$)	$52.4\% \pm 0.4\%$ ($\bar{n} = 0.1996$)	$99.9\% \pm 0.8\%$ ($\bar{n} = 0.2082$)
$\|0,4\rangle$	$99.1\% \pm 2.5\%$ ($\bar{n} = 0.2134$)	$87.6\% \pm 2.2\%$ ($\bar{n} = 0.2082$)	$65.7\% \pm 1.7\%$ ($\bar{n} = 0.2097$)	$99.9\% \pm 2.5\%$ ($\bar{n} = 0.2082$)
$\|0,5\rangle$	$97.8\% \pm 6.2\%$ ($\bar{n} = 0.2076$)	$96.7\% \pm 7.2\%$ ($\bar{n} = 0.2082$)	$71.4\% \pm 4.6\%$ ($\bar{n} = 0.2097$)	$98.6\% \pm 7.2\%$ ($\bar{n} = 0.1983$)
$\|1,2\rangle$	$74.8\% \pm 0.8\%$ ($\bar{n} = 0.2043$)	$18.9\% \pm 0.3\%$ ($\bar{n} = 0.2082$)	$50.3\% \pm 0.2\%$ ($\bar{n} = 0.1997$)	$79.3 \pm 0.8\%\%$ ($\bar{n} = 0.2051$)
$\|2,2\rangle$	$94.5\% \pm 2.2\%$ ($\bar{n} = 0.2088$)	$42.5\% \pm 1.0\%$ ($\bar{n} = 0.2141$)	$50.6\% \pm 1.2\%$ ($\bar{n} = 0.2097$)	$93.8\% \pm 2.3\%$ ($\bar{n} = 0.2051$)
$\|2,3\rangle$	$97.7\% \pm 7.0\%$ ($\bar{n} = 0.2043$)	$76.6\% \pm 4.7\%$ ($\bar{n} = 0.2150$)	$54.8\% \pm 3.7\%$ ($\bar{n} = 0.2097$)	$99.5\% \pm 7.5\%$ ($\bar{n} = 0.1969$)

the analysis from Section 5, i.e. the source operates in the parametric regime and our photon-number states are near single-mode.

6.2 Computation of probability distributions and estimation of errors

Experimental demonstration of two-mode multi-photon HOM interference requires collecting photon-number statistics, which are then compared with theoretical probability distributions. The statistics result from multiple measurements performed with the setup depicted in Fig. 1**B** in the main text. The heralding modes (A & D) inform about the input state fed into the variable BS and together with the output modes are measured by highly efficient photon counting TES detectors. Thus, each measurement results in a 4-tuple consisting of the number of photons registered by TES_{1-4}, denoted as (n_1, n_2, n_3, n_4) and corresponding to photon-number states in modes A–D (*29*). In a single run, the SPDC source produces input Fock states consisting of up to approximately 10 photons with probability governed by the pump power (see the *Materials and Methods* section in the main text). The detectors register all possible values of $n_i \in [0, 10]$,

$i = 1, \ldots, 4$. The automation software stores this data in a database and assigns the number of events to each possible tuple. During a single 400-second run, approx. 10^9 data points are collected.

In order to obtain a photon-number statistics for a given $r = \sin^2 \frac{\theta}{2}$ and input Fock state a post-processing is required. The database is searched for a given pair (n_1, n_4) which determines the two-mode Fock state at the BS input. Then, only records fulfilling the condition $n_1 + n_4 = n_2 + n_3$ are selected as they may correspond to the case of no losses in all paths. For the given (n_1, n_4) the individual probabilities are computed as

$$p_S(k, n_1 + n_4 - k) = \frac{N\left(n_1, k, n_1 + n_4 - k, n_4\right)}{S(n_1, n_4)}$$

where $N(n_1, n_2, n_3, n_4)$ denotes the number of events of registering the given 4-tuple, $S(n_1, n_4) = \sum_{m=0}^{n_1+n_4} N(n_1, m, n_1 + n_4 - m, n_4)$ is the total number of contributing data points and k as well as $n_1 + n_4 - k$ are the photon numbers registered at the BS outputs. The full probability distribution consists of $n_1 + n_4 + 1$ values for k ranging from 0 to $n_1 + n_4$.

For the TES detectors, due to the overlap between the outcomes associated with neighboring photon numbers, an $|n\rangle$ state results in a value of $n \pm 1$, where n is registered with probability over 0.9 and the probabilities of $n - 1$ and $n + 1$ are below 0.1 with $p(n - 1) \gg p(n + 1)$. Therefore, the absolute error of a single measurement $\Delta n = \pm 1$. As the computation of probability is based on $S(n_1, n_4)$ data points, the measurement uncertainty equals

$$\Delta p = \frac{|\Delta n|}{\sqrt{S(n_1, n_4)}} \approx \frac{1}{\sqrt{S(n_1, n_4)}}$$

The data post-processing and error estimation was done with a Python script, which prepared input files for the Asymptote plotting software. The probability distributions for an ideal system were computed with Eq. S88. Factorials and binomial coefficients were approximated with the standard `lgamma(n)` function.

6.3 Realistic theoretical model

Actual experimental results (Fig. 3 in the main text) were compared with an enhanced realistic theoretical model which allowed to assess the imperfections of the system. The model includes the following parameters: average photon numbers at the outputs of both SPDCs, strength of the fiber coupling, losses in heralded and interfering modes as well as efficiencies of individual TES detectors.

The computations are done with 6×6 complex matrices, where the indexes 1-2 correspond to heralded modes and 3-4 to the outputs of the variable beam splitter. The indexes 5-6 are responsible for the losses in modes entering the beam splitter, which are modeled by two additional beam splitters which bring the SPDC outputs B and C to interference with the vacuum state. The TES detectors are described by the probability of detecting n_d photons in a Fock state $|n_{\text{in}}\rangle$, given by the following formula

$$p_{\text{TES}}(n_{\text{in}}, n_d, \eta) = \begin{cases} \binom{n_{\text{in}}}{n_d} (1-\eta)^{n_{\text{in}}-n_d}\, \eta^{n_d} & \text{if } n_d \leq n_{\text{in}} \\ 0 & \text{otherwise} \end{cases} \tag{S118}$$

where η is the efficiency of the detector, additionally decreased to model imperfections in optical signal transfer (e.g. fiber coupling). The distribution in Eq. S118 well models detectors used in the experiment (*20*).

The numerical program was written in the Java programming language and run on a standard PC. It allows to compute output probability distributions $p_S(k, l)$ for given set of model parameters and given readouts at heralded modes (n_1, n_4). The computation results were passed to Python scripts which prepared Asymptote data files to be merged with experimental plots. The computations were performed for the same input Fock states as in Fig. 3 in the main text and mean number of photons equal to 0.2. Then, the program was run for various parameters in order to fit the theoretical distributions to the actual experimental data. The results are presented in Fig. S7.

7 Mapping between qudit and interacting spin-$\frac{1}{2}$-chain quantum computer architectures

Any state of a d-level qudit can be encoded in a chain of d qubits where only one qubit is excited at a time, i.e. using the single excitation basis $|1, 0, \ldots, 0\rangle$, $|0, 1, \ldots, 0\rangle$, etc. The XY Heisenberg model maps the next-neighbor interaction in the chain to the qudit rotation discussed in the main text.

7.1 XY model

Let us consider an interacting chain of N qubits governed by the following Hamiltonian

$$H_{XY} = \sum_{n=1}^{N} \frac{J_n}{2} \left[\sigma_n^x\, \sigma_{n+1}^x + \sigma_n^y\, \sigma_{n+1}^y\right] \tag{S119}$$

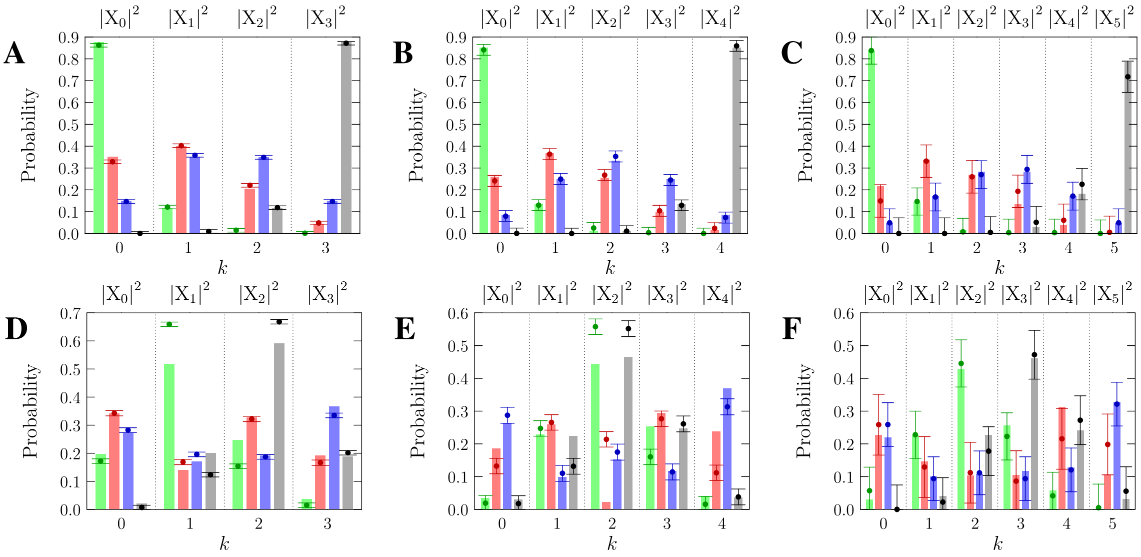

Fig. S7. Photon number statistics resulting from Fock state $|l, S-l\rangle$ interference. (**A**) $|0,3\rangle$, (**B**) $|0,4\rangle$, (**C**) $|0,5\rangle$, (**D**) $|1,2\rangle$, (**E**) $|2,2\rangle$, (**F**) $|2,3\rangle$. The BS reflectivities are $r = 0.05$ (green), 0.2 (red), 0.5 (blue) and 0.95 (gray). Vertical bars represent theoretical values computed for a realistic system, while dots are values determined in experiment – the probabilities of detecting $|k\rangle$ and $|S-k\rangle$ photons behind the BS. The parameters of computation: mean number of photons generated by SPDC equal to 0.2, TES detection efficiency – 0.9, fiber coupling – 0.7 and overall losses in the system – 50%. The states (**A**)-(**C**) encode sequences $(x_0 = 1, x_1 = 0, \ldots, x_S = 0)$, while in (**D**) – $(0,1,0,0)$, (**E**) – $(0,0,1,0,0)$, (**F**) – $(0,0,1,0,0,0)$, respectively. The measured probabilities set their QKTs $(|\mathrm{X}_0|^2, |\mathrm{X}_1|^2, \ldots, |\mathrm{X}_S|^2)$, $|X_k|^2 = |\sum_{l=0}^{S} \mathcal{A}_S^{(r)}(k,l) \cdot x_l|^2$ of fractionality $\alpha = 0.28$ (green), 0.60 (red), 1.00 (blue) and 1.72 (gray).

where σ_n^x, σ_n^y, σ_n^z are the Pauli operators acting on the nth qubit and J_n denote couplings between neighboring qubits in the chain.

We first note that a spin-$\frac{N-1}{2}$ particle corresponds to an N-qubit chain with relabeled basis vectors as $|m\rangle$, where $m = -\frac{N-1}{2} + n - 1$ (*40*). The N-qubit Hilbert space is of dimension 2^N. Let us restrict H_{XY} to the N-dimensional single-excitation subspace of this system. This subspace is spanned by the basis vectors $|n\rangle$, $n = 1, \ldots, N$, corresponding to spin configurations in which all spins are "down" apart from just one spin at the vertex n which is "up", i.e. by the eigenstates of the $\sigma_{tot}^z = \sum_i \sigma_i^z$ operator. Then H_{XY} is identical to the Hamiltonian of a spin-$\frac{N-1}{2}$ particle $H = \lambda S_x$, where λ is a constant. Here $J_n = \frac{\lambda}{2}\sqrt{n(N-n)}$. This particular form of J_n allows us to link the XY with the BS interaction. The BS infinitesimal evolution turn the input state $|l, S-l\rangle$ into the superposition

$$H_{BS}|l, S-l\rangle = q_{l,l-1}\,|l-1, S-l+1\rangle + q_{l,l+1}\,|l+1, S-l-1\rangle \tag{S120}$$

with the amplitudes

$$q_{l,l+1} = \frac{\sqrt{(l+1)(S-l)}}{2} \tag{S121}$$

The amplitudes reproduce J_n for $N = S+1$, $n = l+1$ and $\lambda = 1$.

7.2 Example: quantum annealing processor

A Hamiltonian describing quantum annealing processor based on N interacting qubits reads

$$H_S(s) = \mathcal{E}(s)H_P - \frac{1}{2}\sum_i \Delta(s)\sigma_i^x, \quad i = 1, \ldots, N \tag{S122}$$

where s denotes time ($s = t/t_f$, $t \in [0, t_f]$), $\mathcal{E}(s)$ and $\Delta(s)$ are the transverse and longitudinal energies, respectively. H_P is a dimensionless Hamiltonian

$$H_P = -\sum_i h_i\,\sigma_i^z + \sum_{i<j} J_{ij}\,\sigma_i^z\,\sigma_j^z \tag{S123}$$

where biases h_i and couplings J_{ij} encode a particular optimization problem. Quantum annealing starts with setting $\Delta \gg \mathcal{E}$, then Δ is reduced and $\mathcal{E}$ is increased until $\mathcal{E} \gg \Delta$ and $H_S \approx H_P$.

Thus, initially the qubit register is prepared in an eigenstate of the $\sigma_{tot}^x = \sum_i \sigma_i^x$ operator and then the following evolution $\sum_{i<j} J_{ij}\,\sigma_i^z\,\sigma_j^z$ is applied (for simplicity we assume $h_i = 0$). If we now take $J_{ij} = J_n$ for two neighboring qubits and $J_{ij} = 0$ otherwise, we will reproduce the evolution in the XY model, where the register is initially in the eigenstate of σ_{tot}^z and evolution takes place in the orthogonal subspace $\sum_n \frac{J_n}{2}\left[\sigma_n^x\,\sigma_{n+1}^x + \sigma_n^y\,\sigma_{n+1}^y\right]$.

7.3 How to perform the QKT of MRI data?

The MRI frequency data form a matrix of complex coefficients $\{f_{x,y}\}$, $x, y = 1, \ldots, N$, and their processing requires a two-dimensional QKT. Thus, the input data have to be encoded in a 2D quantum superposition with $\{f_{x,y}\}$ defining its amplitudes For a spin chain implementation this could be the following encoding

$$|\Psi_{in}\rangle = \sum_{i,j=1}^{N} f_{i,j} \, |0_1, \ldots, 1_i, \ldots, 0_N\rangle \, |0_1, \ldots, 1_j, \ldots, 0_N\rangle \tag{S124}$$

Here a long chain of spins is divided into two subchains, and the operations on them are performed independently.
